# Defective Viral Genomes Arising *In Vivo* Provide Critical Danger Signals for the Triggering of Lung Antiviral Immunity

**DOI:** 10.1371/journal.ppat.1003703

**Published:** 2013-10-31

**Authors:** Karla Tapia, Won-keun Kim, Yan Sun, Xiomara Mercado-López, Emily Dunay, Megan Wise, Michael Adu, Carolina B. López

**Affiliations:** Department of Pathobiology, School of Veterinary Medicine, University of Pennsylvania, Philadelphia, Pennsylvania, United States of America; Yale University School of Medicine, United States of America

## Abstract

The innate immune response to viruses is initiated when specialized cellular sensors recognize viral danger signals. Here we show that truncated forms of viral genomes that accumulate in infected cells potently trigger the sustained activation of the transcription factors IRF3 and NF-κB and the production type I IFNs through a mechanism independent of IFN signaling. We demonstrate that these defective viral genomes (DVGs) are generated naturally during respiratory infections *in vivo* even in mice lacking the type I IFN receptor, and their appearance coincides with the production of cytokines during infections with Sendai virus (SeV) or influenza virus. Remarkably, the hallmark antiviral cytokine IFNβ is only expressed in lung epithelial cells containing DVGs, while cells within the lung that contain standard viral genomes alone do not express this cytokine. Together, our data indicate that DVGs generated during viral replication are a primary source of danger signals for the initiation of the host immune response to infection.

## Author Summary

In infections with viruses well adapted to the host virus-encoded proteins that delay the cellular response allow the virus to replicate to high titers prior to host intervention. The mechanisms overcoming viral evasion of the immune system and leading to the production of the primary antiviral cytokine IFNβ are not well established. Here, we demonstrate that truncated forms of viral genomes that are generated *in situ* during virus replication are a primary source of danger signals for the initiation of the host immune response to respiratory viral infections *in vivo*. Defective viral genomes (DVGs) are able to function as triggers of the immune response even in the absence of type I IFN signaling and are strong triggers of the host response to infection while overcoming viral antagonism.

## Introduction

The recognition of virus-specific pattern associated molecular patterns (PAMPs) is a pivotal event in the initiation of the host innate response to infection. In recent years, it has been established that most viral danger signals are derived from oligonucleotide structures exposed during the replication of the viral genomes [1], [2], [3], [4], [5], [6]. However, most viruses produce proteins that antagonize and effectively delay signaling by the primary viral oligonucleotide sensor molecules retinoic acid inducible gene I (RIG-I) and melanoma differentiation–associated gene 5 (MDA5), allowing the virus to replicate to high titers and produce large amounts of danger signals prior to host intervention [7], [8]. It is currently unclear how the host immune response overcomes viral evasion to initiate a protective antiviral response.

Defective viral genomes (DVGs) arise when the viral polymerase loses processivity during virus replication at high titers, thereby generating truncated versions of the viral genome that contain deletions and/or complementary ends (the later known as copy-back or snap-back genomes) [9], [10]. DVGs with the ability to interfere with standard virus replication were first described by Von Magnus in the early 1950s as the genomes of incomplete forms of influenza virus called defective interfering (DI) viral particles [11]. DVGs have been identified in multiple distinct viral families when the viruses are grown in the laboratory at high multiplicity of infection and span a broad range of hosts, from plants to mammals [12]. Importantly, DVGs are found in patients infected with hepatitis A [13], hepatitis B [14], [15], hepatitis C [16], HIV [17], dengue virus [18], and influenza virus [19]. However, the biological role of DVGs in the context of natural infections is not well understood.

We and others have shown that stocks of Sendai virus (SeV) with a high content of copy-back DVGs with interfering activity trigger enhanced production of cytokines *in vitro* and more potently induce antigen presentation by mouse and human dendritic cells than do virus stocks lacking this kind of DVGs [20], [21], [22], [23], [24], [25]. Our group has also demonstrated that in contrast to standard viral genomes, SeV copy-back DVGs induce the expression of MDA5 and of a number of other interferon-stimulated genes in the absence of type I IFN positive feedback [23], [26], [27]. Remarkably, SeV copy-back DVGs show this potent *in vitro* stimulatory activity even in the presence of functional viral encoded antagonists of the host response [23], [24]. Here, we demonstrate that DVGs that trigger a robust activation of the transcription factors IRF3 and NF-κB accumulate at a high rate in infected cells becoming the main source of viral PAMPs. These DVGs arise naturally during acute respiratory viral infections in mice and provide essential stimuli for the initiation of the antiviral innate immune response in the lung. These data demonstrate the generation of DVGs *in vivo* during acute respiratory viral infections and suggest a critical role of these kinds of viral genomes in determining the quality of the host response to infection.

## Results

### SeV copy-back DVGs trigger a robust and sustained activation of IRF3 and NF-κB independent of type I IFN feedback

**Figure 1:**
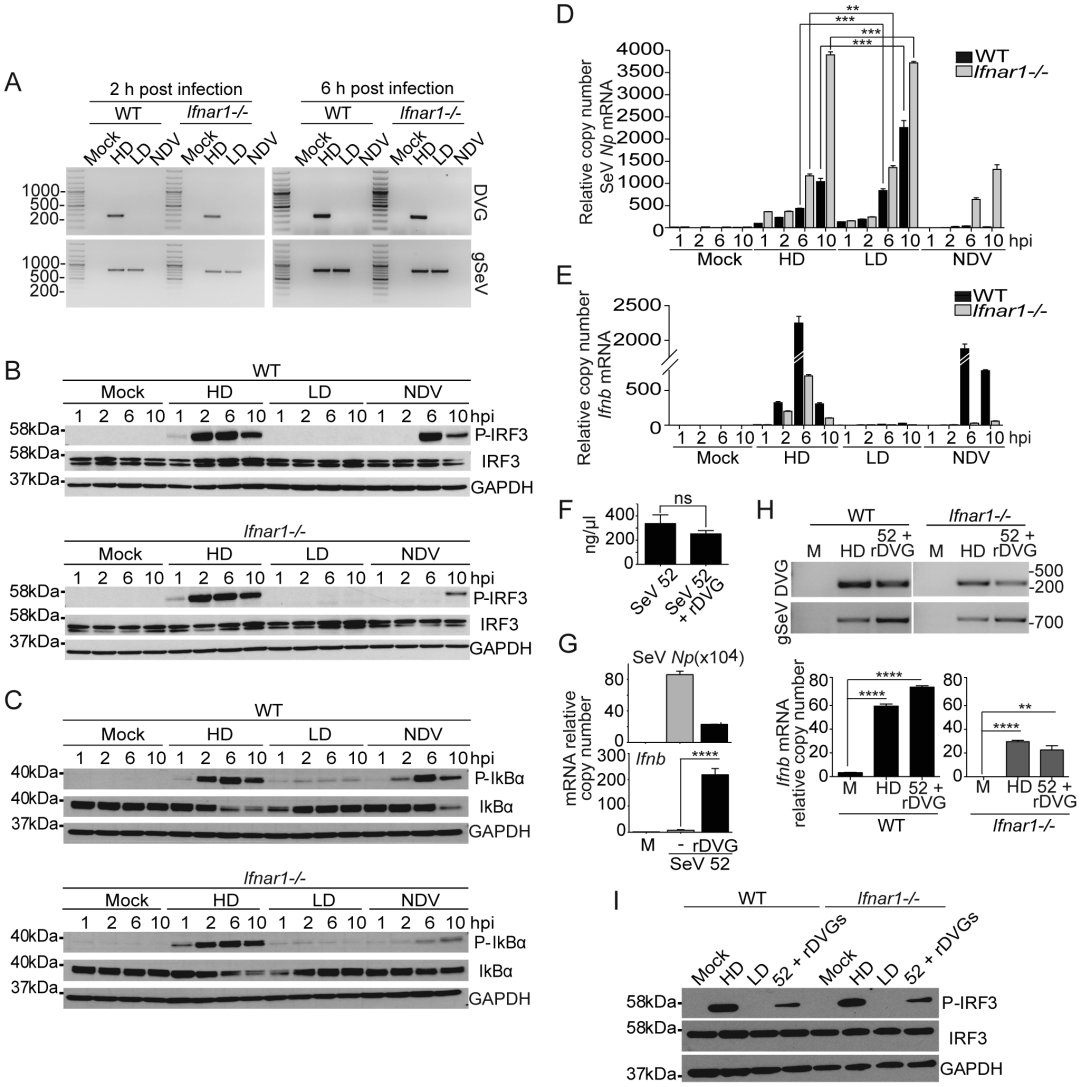
Robust and sustained activation of IRF3 and NF-κB independently of type I IFN feedback in response to SeV DVGs. WT and *Ifnar1^−/−^* BMDCs were infected with SeV Cantell HD (HD), SeV Cantell LD (LD), or NDV (moi = 1.5 TCID_50_/cell). (**A**) Total RNA was extracted at 2 and 6 h post-infection and analyzed for the presence of DVGs and standard virus genome (gSeV) by PCR. Position of base pair size reference markers is indicated in each gel. Whole cellular extract was prepared and examined for phosphorylation of (**B**) IRF3 and (**C**) IκBα by western blot at the indicated time points. Total RNA was extracted from the infected cells and the expression of (**D**) SeV *Np* or NDV *Hn* mRNA and (**E**) *Ifnb* was monitored by RT-qPCR. (**F**) Total RNA content on equivalent infectious doses of SeV strain 52 or SeV 52 containing rDVGs. (**G**) LLC-MK2 cells infected with a moi = 5 TCID_50_/cell SeV 52 alone or in the presence or a recombinant (r)DVG. Expression of viral *Np* and *Ifnb* mRNA was determined by RT-qPCR 18 h after infection. (**H**) WT and *Ifnar1* ^−/−^ mouse embryo fibroblasts were infected as indicated. Presence of WT or recombinant (r)DVG in the infected cells was determined by PCR 6 h after infection. Expression of the viral *Np* and *Ifnb* mRNA was determined by RT-qPCR 6 h after infection. (**I**) Whole cell extracts were analyzed for P-IRF3 by western blot. IRF3 and GAPDH are shown as controls. Gene expression is shown as copy number relative to the housekeeping genes *Tuba1b* and *Rps11*. Error bars indicate the standard deviation of triplicate measurements in a representative experiment (**p<0.01, ***p<0.001, ****p<0.001 by one-way ANOVA with Bonferroni post hoc test).

To further investigate the cellular mechanisms responsible for the efficient activation of the antiviral response by SeV DVGs, we evaluated the phosphorylation of transcription factors that are critical for the expression of type I IFNs in cells infected with equivalent amounts of infectious particles of a SeV strain Cantell stock containing high levels of copy-back DVGs (SeV Cantell HD) or with SeV Cantell depleted of DVGs (SeV Cantell LD). Virus stocks were prepared from the same parental virus and their content of DVGs was determined by calculating the ratio of infectious particles to total particles (ratios are specified in the material and methods section). In addition, copy-back DVGs of these stocks were identified by PCR. One predominant copy-back genome was present in cells infected with SeV Cantell HD (amplicon of 278 bp), while no copy-back defective genome was detected in cells infected with SeV Cantell LD up to six hours after infection (Figs. 1A and S1). Cloning and sequencing of the 278 nt long amplicon confirmed that it corresponded to a previously described SeV Cantell copy-back DVG of 546 nt in length (DVG-546) [28]. Phosphorylation of IRF3 and of the NF-κB repressor IκBα in response to SeV Cantell HD occurred rapidly and was sustained even in type I IFN receptor KO cells (*Ifnar1^−/−^*) (Fig. 1B and C), while no phosphorylation of IRF3 or IκBα was observed for up to ten hours post-infection with SeV Cantell LD despite equivalent or higher expression of the viral protein *Np* (Fig. 1D). Corresponding with the strong activation of transcription factors, *Ifnb* mRNA was expressed in *Ifnar1^−/−^* cells infected with SeV Cantell HD (Fig. 1E). In contrast, type I IFN signaling was required for the cellular response to Newcastle disease virus (NDV), an avian virus that only partially inhibits the type I IFN pathway, triggering the expression of type I IFN and other cytokines in the absence of DVGs.

To further validate the role of SeV copy-back DVGs as triggers of type I IFN-independent antiviral responses, we cloned DVG-546 under the control of the T7 polymerase promoter and used this construct to prepare a SeV stock containing a single recombinant DVG (rDVG). For this purpose we used SeV strain 52 that normally does not produce highly immunostimulatory copy-back DVGs [24]. Equivalent infectious units of SeV 52 and SeV 52 plus rDVGs had similar levels of total RNA (Fig. 1F) but infection with virus containing rDVGs strongly induced the antiviral response while virus that lacked DVGs did not (Fig. 1G) confirming the DVG immunostimulatory activity. In addition, presence of rDVGs significantly reduced the expression of SeV *Np* mRNA, demonstrating their strong interfering capacity (Fig. 1G). Notably, mouse embryo fibroblasts lacking the type I IFN receptor expressed *Ifnb* mRNA in response to SeV 52 containing rDVG (Fig. 1H) and virus containing rDVGs triggered IRF3 phosphorylation independently of type I IFN feedback (Fig. 1I), mirroring the response to SeV Cantell HD. Altogether, this evidence conclusively shows that SeV copy-back DVGs confer potent immunostimulatory ability to SeV stocks, independent of type I IFN feedback. Notably, potent *Ifnb* mRNA expression in response to SeV DVGs was independent of IRF1, IRF5, and IRF8 while only partially dependent on IRF7 (Fig. S2). This response was maintained in a variety of cell types (Fig. S3).

### DVGs accumulate at a high rate in infected cells and are a primary source of pathogen associated molecular patterns that trigger RLR signaling

**Figure 2:**
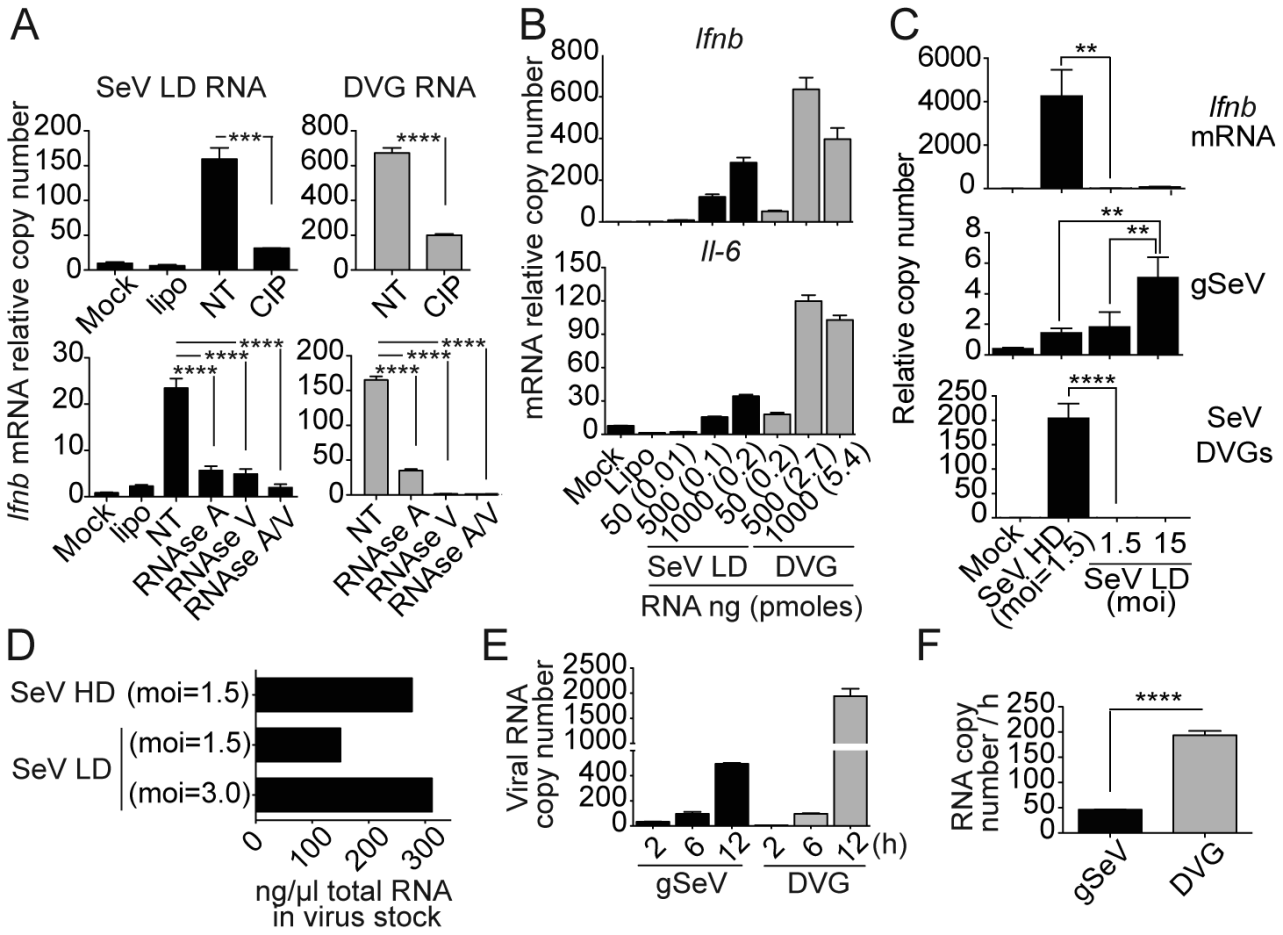
DVGs accumulate at a high rate in infected cells and are associated with the induction of the antiviral responses. (**A**) SeV Cantell LD RNA and DVG RNA were extracted from SeV Cantell LD stocks or *in vitro* transcribed DVG RNA respectively. RNAs were treated with calf intestinal phosphatase, RNase A, RNase V1, and/or RNase A/V1. LLC-MK2 cells were transfected with 250 ng of control or treated RNAs and analyzed 4 h after transfection by RT-qPCR. (**B**) LLC-MK2 cells were transfected with the indicated doses of genomic RNA and DVG RNA. After 4 h, total RNA was extracted and expression of *Ifnb* and *Il-6* was determined by RT-qPCR. (**C**) WT MEF cells were infected with SeV Cantell HD or SeV Cantell LD at the indicated mois. After 6 h, total RNA was extracted and the quantities of *Ifnb* mRNA, gSeV, and DVG were determined by RT-qPCR. (**D**) Total RNA was directly quantitated from SeV Cantell HD or LD solutions prepared to have the indicated infectious units. (**E**) WT MEF cells were infected with SeV Cantell HD at a moi of 3 TCID_50_/cell and total RNA was analyzed by RT-qPCR at the indicated times post-infection. (**F**) Rate of gSeV and DVG replication calculated at 12 h post-infection. Gene expression is shown as copy number relative to the housekeeping genes *Tuba1b* and *Rps11*. Error bars indicate the standard deviation of triplicate measurements in a representative experiment (**p<0.01, ***p<0.001, ****p<0.001 by one-way ANOVA with Bonferroni post hoc test (A and C); ****p<0.001 by Unpaired, two tailed, t student test (F)).

To determine whether standard viral genomes and DVG RNAs have distinct intrinsic properties that explain their differential immunostimulatory activities, we compared naked RNA purified from a stock of SeV Cantell LD with *in vitro* transcribed DVG-546. RNAs were transfected into cells before or after treatment with phosphatase or with RNase A that cleaves 3′ of single stranded C and U residues, and/or RNase V1 that cleaves base paired nucleotides. Both genomic RNA and DVG RNA were susceptible to treatment with phosphatase, as well as to treatment with RNases (Fig. 2A), corresponding with the literature that demonstrates a crucial role for 5′-triphosphate-RNA in the induction of type I IFNs. While transfected DVGs induced stronger expression of *Ifnb* than an equivalent concentration of gSeV (Fig. 2A), transfection of equivalent molar amounts of genomic and DVG RNA resulted in higher immunostimulatory activity of genomic RNA compared to DVG RNA (Fig. 2B), demonstrating that SeV LD RNA can strongly trigger the host response to infection when delivered naked into the cells.

Paradoxically, cells infected with SeV Cantell LD alone failed to induce strong type I IFN production even when used at a 10 times higher infectious dose than SeV Cantell HD (Fig. 2C). Although the amount of gSeV RNA was significantly higher in cells infected with an moi of 15 of SeV Cantell LD compared with ten times less SeV Cantell HD at 6 h post-infection (Fig. 2C), DVGs were only detected in cells infected with SeV Cantell HD, confirming a strong correlation between the presence of DVGs in the infected cells and the induction of the host response to infection. To determine whether the amount of total input viral RNA affected the immunostimulatory activity of SeV Cantell LD and HD, we measured the RNA content in equivalent infectious doses of these stocks. SeV Cantell HD had less than two fold higher the amount of total RNA than SeV Cantell LD and total RNA levels were equivalent between SeV Cantell LD and HD when LD was at twice the infectious dose (Fig. 2D). Thus, differences in the net input amount of viral RNA cannot explain the more than >1000 fold difference in the expression of *Ifnb* mRNA between cells infected with equivalent infectious doses of SeV Cantell LD and HD.

DVGs have an increased rate of replication compared to standard viral genomes due to their shorter size and promoter properties [29]. To determine whether DVGs replicate faster than gSeV, we calculated the rate of replication of gSeV and DVGs in cells infected with SeV Cantell HD. Although at an early time point more copies of gSeV than DVGs were detected in the cells, DVGs dominated by 12 h post-infection (Fig. 2E) accumulating at a 4 times faster rate than gSeV (Fig. 2F). These data demonstrate that DVGs rapidly surpass the number of gSeV in infected cells, providing large quantities of pathogen associated molecular patterns.

Supporting previous observations that DVGs from SeV stimulate the cellular antiviral response through signaling by RIG-I like receptors (RLRs) [1], [23], [24], the essential RLR adaptor protein mitochondrial antiviral signaling protein (MAVS) was required for the activation of the transcription factors IRF3 and NF-κB and for expression of numerous antiviral and pro-inflammatory molecules upon infection with SeV Cantell HD. In contrast, MAVS was not required for the response to herpes simplex virus, which can trigger the host response independently of RLRs (Fig. S4). In addition, only DVG RNA, but not standard viral genomes, could be amplified from endogenous RIG-I and MDA5 complexes immunoprecipitated from infected cells (Fig. S4C), supporting published evidence that DVGs bind to RIG-I preferentially over the standard viral genomes in infected cells [1]. As predicted, association of DVGs with RLRs correlated with type I IFN induction, but not with the level of virus replication (Fig. S4D). Importantly, in addition to the primary role of RIG-I in the response to SeV DVGs, MDA5 participates in the induction of type I IFN in primary mouse lung fibroblasts infected with SeV HD (Fig. S4E), similar to what we have observed in DCs [23], [27].

Overall, these data demonstrate that DVGs are produced in the infected cells at a higher rate than genomic RNA and that DVGs are the predominant ligands for both RIG-I and MDA5 during SeV infection.

### DVGs generated *in situ* during infection have strong immunostimulatory activity

**Figure 3:**
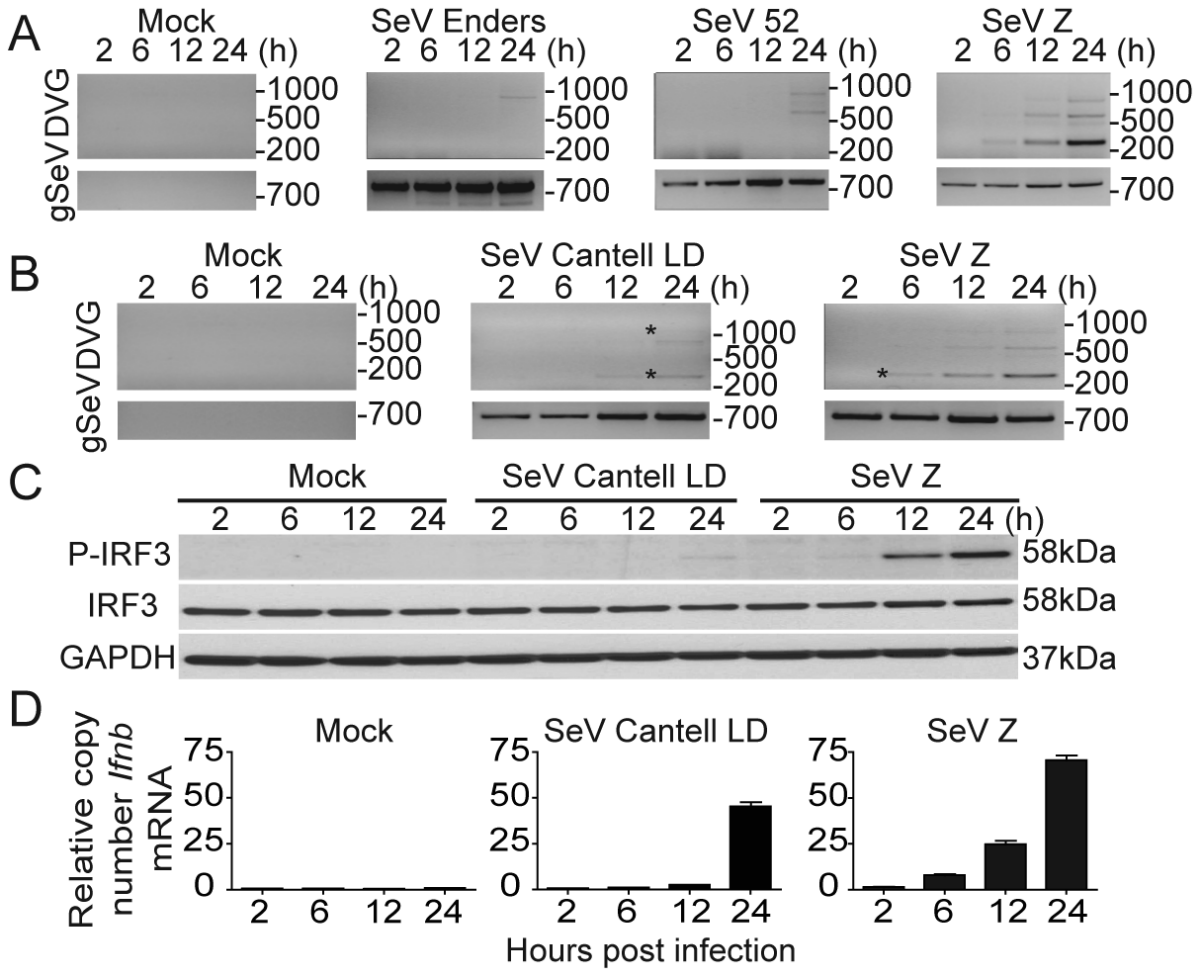
SeV copy-back DVGs generated *in situ* during infection have a strong stimulatory ability. (**A**) TC-1 cells were either mock-infected or infected with a moi of 1.5 TCID_50_/cell of SeV strains Enders, 52, or Z. Total RNA was extracted at 2, 6, 12, and 24 h post-infection and analyzed for the presence of DVGs and standard virus genome (gSeV) by PCR. (**B**) BMDCs were infected with SeV strain Z or SeV Cantell LD and RNA was extracted at 2, 6, 12, and 24 h post-infection and analyzed for the presence of DVGs and gSeV. (**C**) Immunoblot of phosphorylated IRF3 in whole cell extracts from infected BMDCs and (**D**) *Ifnb* mRNA expression in infected BMDCs as determined by RT-qPCR. Gene expression is shown as copy number relative to the housekeeping genes *Tuba1b* and *Rps11*. Sequences from bands labeled with a star can be found in Fig. S5. Position of base pair size reference markers is indicated in each gel.

Based on the potent ability of SeV stocks containing a high content of copy-back DVGs to induce the host response to infection *in vitro* [23], [24], [25], [28] (Fig. 1) and on our prior reports of strong host responses to DVGs regardless of the presence of functional virus-encoded antagonists [23], [24], we hypothesized that DVGs that arise *in situ* during viral infections provide essential stimuli to initiate an antiviral immune response. To test this hypothesis, we first determined if SeV strains that accumulate copy-back DVGs early in infection induced faster *Ifnb* mRNA expression *in vitro* than viruses with delayed DVG accumulation. For these experiments we used SeV preparations that did not show immunostimulatory activity or evidence of copy-back DVG accumulation by 2 h post-infection and all the viruses were used at a multiplicity of infection of 1.5 TCID_50_/cell. While standard viral genomes of all the different SeV strains used were detected at all tested time points, copy-back DVGs of different sizes were detected starting at 6 h post-infection in cells infected with SeV Z and at later time points in cells infected with SeV 52, Enders, or Cantell LD in both murine lung epithelial cells (TC-1) and bone marrow-derived dendritic cells (BMDCs) (Fig. 3A and B and data not shown). Sequences of the starred PCR products confirming the amplification of copy-back DVGs are shown in Fig. S5. Remarkably, accumulation of DVGs was directly associated with phosphorylation of IRF3 (Fig. 3C) and with the expression of *Ifnb* mRNA (Fig. 3D), demonstrating that standard viral genomes alone are not sufficient to initiate this response during infection *in vitro* and strongly supporting a unique ability of naturally arising DVGs to initiate the cellular antiviral response.

### DVGs diminish SeV virulence in mice

**Figure 4:**
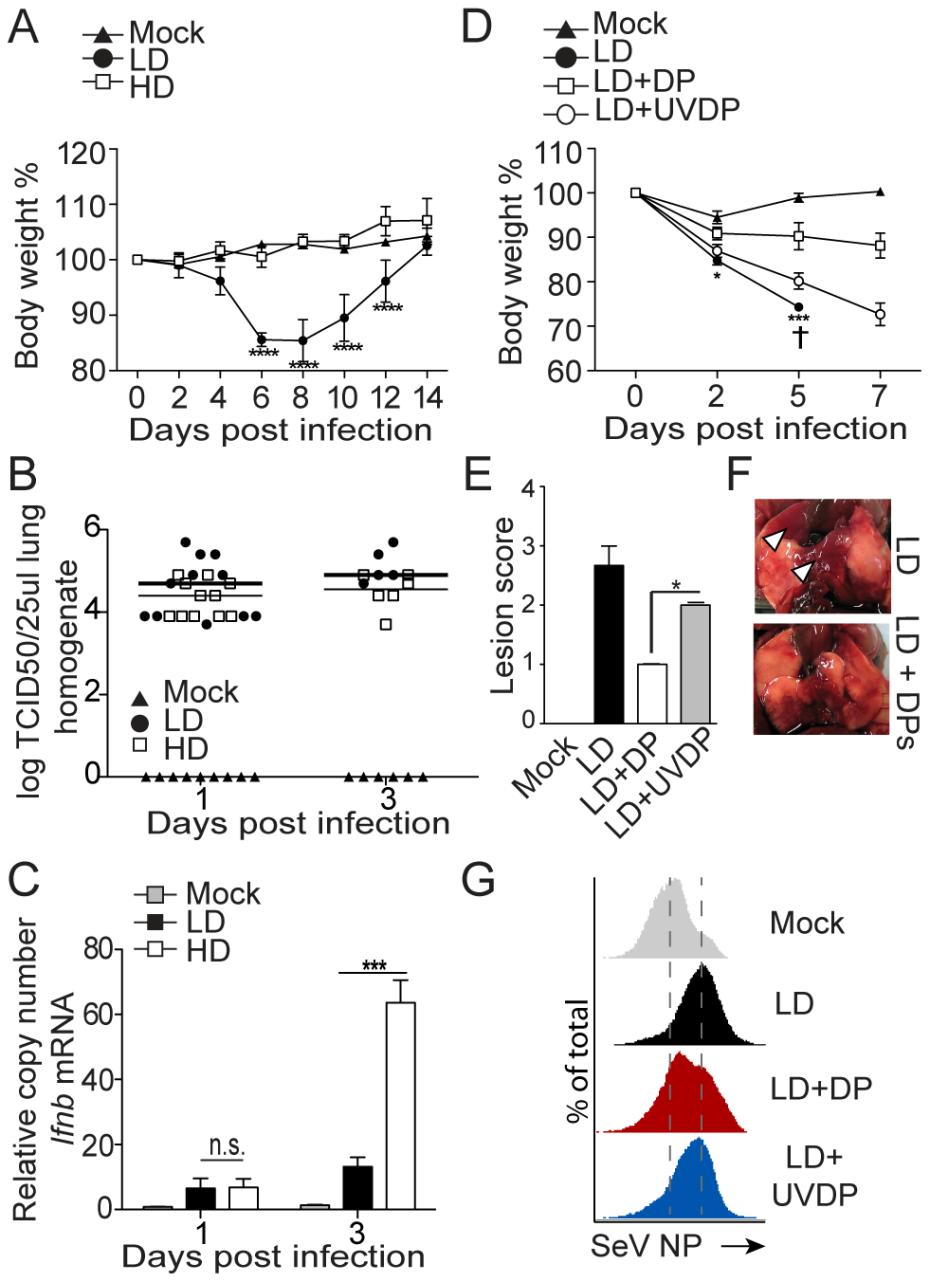
SeV DVGs reduce virulence *in vivo*. (**A–C**) Mice were infected with 10^5^ TCID_50_/mouse of SeV Cantell HD (HD) or SeV Cantell LD (LD). (**A**) Weight loss (***p<0.001, ****p<0.0001; Two-way ANOVA with Bonferroni's post hoc test), (**B**) Virus titers in the lung, (n = 11 for day 1, n = 6 for day 3), and (**C**) expression of *Ifnb* mRNA by RT-qPCR. Gene expression is shown as copy number relative to the housekeeping genes *Tuba1b* and *Rps11* (***p<0.001, Unpaired, two tailed, t student test). (**D–F**) Mice were infected with 10^4^ TCID_50_/mouse SeV Cantell LD alone, in the presence of 5,000 HA Units/mouse purified defective particles (DPs) or in the presence of UV-inactivated DPs (UVDP). Mice received DPs or UVDPs immediately following virus inoculation. (**D**) Weight loss, (†, mice sacrificed due to severe weight loss; ***p<0.001, ****p<0.0001; Two-way ANOVA with Bonferroni's post hoc test), (**E**) lung lesion score at day 7 post-infection (n = 3)(*p<0.05, Mann-Whitney test). (**F**) Photos of the lung of a representative mouse at day 7 post-infection. Arrowhead indicates areas of lesions. (**G**) Lungs from mice infected with SeV Cantell LD alone, or in the presence of DPs or UVDPs were analyzed by flow cytometry for the expression of the SeV NP protein.

To evaluate the impact of DVGs during SeV infection *in vivo*, we infected mice with SeV Cantell HD or LD. Mice infected with SeV Cantell HD showed diminished morbidity than mice infected with the same infectious dose of SeV Cantell LD (Fig. 4A) despite equivalent levels of virus in the lungs at early times post-infection (Fig. 4B), agreeing with reports of reduced virulence in virus stocks with a high content of DVGs [30], [31], [32], [33], [34], [35]. Reduced virulence of SeV Cantell HD was associated with a stronger stimulation of the host antiviral response as shown by the expression of *Ifnb* mRNA (Fig. 4C). To conclusively demonstrate the role of DVGs in diminishing virulence *in vivo*, we co-infected mice with SeV Cantell LD and purified viral particles containing DVGs (defective particles; DPs). Confirming their critical role, DVGs reduced the pathogenicity of SeV Cantell LD in mice, while UV-inactivated DP particles did not provide significant protection (Figs. 4D–F). Interestingly, infection in the presence of DPs resulted in reduced expression of SeV NP protein in the lung at day 7 post-infection, suggesting that in this system, DPs reduce virulence by interfering with virus replication.

### Copy-back DVGs generated in the lung promote the expression of antiviral cytokines

**Figure 5:**
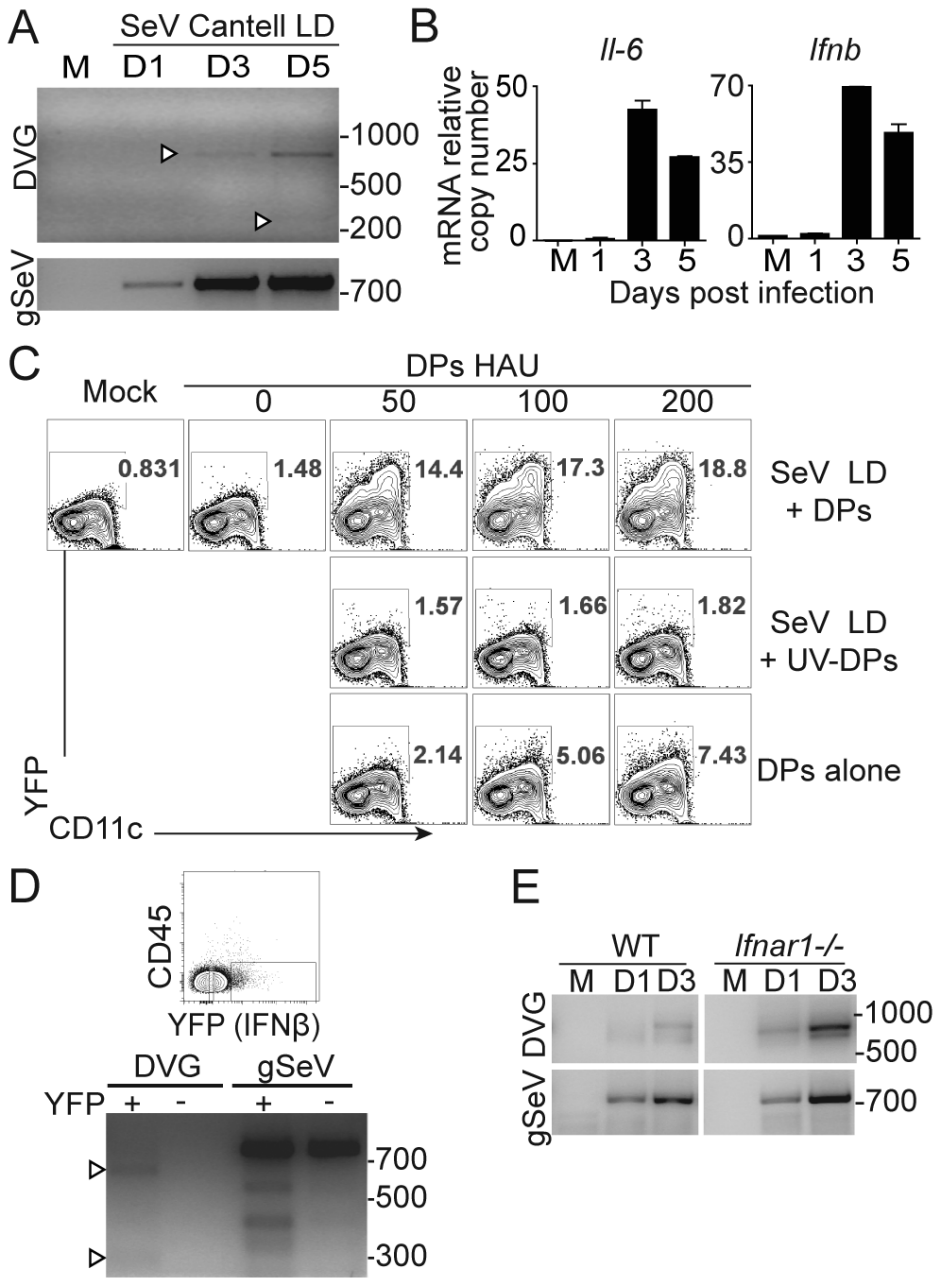
SeV copy-back DVGs are generated in the lung during infection. (**A**) WT mice were infected with 10^4^ TCID_50_/mouse of SeV Cantell LD or mock infected (M) and total RNA from lungs was analyzed for copy-back DVGs and standard viral genomes (gSeV) by PCR. (**B**) Expression of *Ifnb* mRNA in whole lung homogenates was analyzed by RT-qPCR. Gene expression is shown as copy number relative to the housekeeping genes *Tuba1b* and *Rps11*. (**C**) BMDCs from IFNβ-YFP mice were infected with a moi of 1.5 TCID_50_/cell of SeV Cantell LD alone, in the presence of purified DPs or UV-inactivated DPs, or with DPs alone and analyzed by flow cytometry 6 h post-infection. (**D**) CD45^−^ cells were isolated from IFNβ-YFP mice 3 days after infection with SeV Cantell LD and sorted based on YFP expression. Total RNA from the YFP positive and negative fractions was extracted and analyzed for copy-back DVGs and standard viral genomes by PCR. (**E**) Lungs from WT and *Ifnar1* ^−/−^ mice infected with 10^4^ TCID_50_/mouse SeV Cantell LD or mock-infected (M) were analyzed for DVGs and standard viral genomes (gSeV) by PCR. M: mock infected. Position of base pair size reference markers is indicated in each gel.

To determine whether immunostimulatory DVGs were generated *in situ* in the lung during infection, we infected mice with SeV Cantell LD, and we followed the appearance of copy-back DVGs in the lung by PCR. SeV copy-back DVGs were detected in whole lung homogenates at the time of high viral replication (Fig. 5A). Notably, upon infection with SeV Cantell LD, a copy-back DVG of high molecular weight was detected at day 3 post-infection in the lung, while a DVG of low molecular weight (amplicon of 278 bp) that predominates in the parent stock of SeV Cantell HD (Fig. 1A) was only detectable at day 5 post-infection. Copy-back DVGs also appeared in the lung of mice infected with SeV 52 (Fig. S6), showing that DVGs naturally arise during infection *in vivo* independent on the virus strain. Interestingly, accumulation of copy-back DVGs during infection with SeV Cantell LD was associated with the expression of *Ifnb* and *Il-6* mRNA in the lung (Fig. 5B).

To determine whether DVGs were necessary for the expression of antiviral cytokines *in vivo*, we took advantage of IFNβ-YFP reporter mice. To demonstrate that YFP expression serves as readout for DVG activity, we first infected BMDCs prepared from IFNβ-YFP reporter mice with SeV Cantell LD alone, or together with increasing doses of purified DPs. As shown in Fig. 5C, at 6 h post-infection, YFP was expressed only in the presence of DPs and in a dose-dependent manner, and the YFP expression was lost when UV-treated DPs were used. DPs alone were also able to induce YFP in a dose-dependent manner, albeit at much lower levels than during co-infection with SeV. These data agree with our previous reports that demonstrate that the immunostimulatory activity of DPs is greatly amplified during DVG replication by the cognate polymerase provided by co-infecting SeV [23] and validate the IFNβ-YFP reporter system as a readout for DVG activity.

We then infected IFNβ-YFP reporter mice with SeV Cantell LD and analyzed viral genomes in YFP^+^ cells. We focused our analysis on the CD45^−^ (non-hematopoietic) cellular fraction of the lung as SeV replicates predominantly in the lung epithelium [36]. Although full-length viral genomes were detected in both YFP^+^ and YFP^−^ CD45^−^ populations sorted three days after infection, DVGs were only found in YFP^+^ cells (Fig. 5D), suggesting that the presence of DVGs promotes IFNβ production in response to virus infection *in vivo*. Together, these findings show that DVGs are normally generated *in situ* in the lung during respiratory infection with SeV, and that their accumulation is associated with the expression of IFNβ in the lung.

### SeV copy-back DVGs are generated in the lung independently of type I IFN signaling

To determine whether type I IFNs produced early upon infection promoted the generation of DVGs in the lung, we infected wild type or type I IFN receptor deficient mice (*Ifnar1* ^−/−^) with SeV Cantell LD and analyzed the lungs at different times post-infection. As shown in Fig. 5E, DVGs accumulated in the lung at a higher rate in mice unable to respond to type I IFNs compared with wild type mice, corresponding with the predicted enhanced rate of virus replication in the lack of type I IFN signaling and demonstrating that type I IFNs are not required for the generation of SeV copy-back DVGs *in vivo*.

### DVGs are generated in the lung during influenza A (IAV) infection

**Figure 6:**
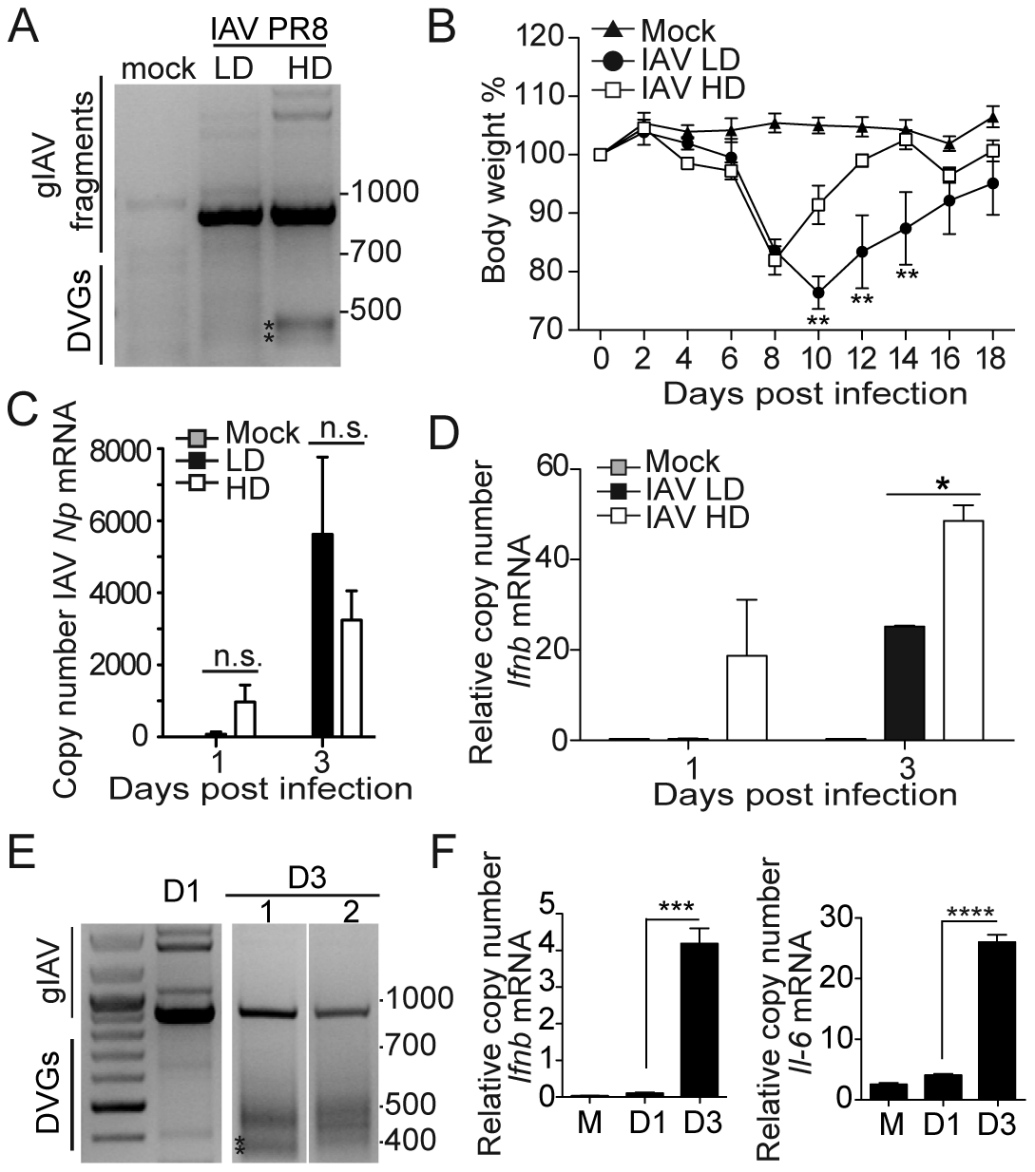
IAV deletion DVGs reduce virus virulence and are generated *in vivo* during infection. (**A**) PCR of standard and defective genomes in BMDCs infected for 6 h with a moi of 1.5 TCID_50_/cell IAV PR8 HD or LD. PCR strategies and sequences of starred product are shown in Fig. S7. (**B**) Weight loss of mice infected with 100 TCID_50_ IAV PR8 HD or LD (n = 5) (**p<0.01; Two-way ANOVA with Bonferroni's post hoc test). (**C**) Quantification of IAV *Np* mRNA and (**D**) *Ifnb* mRNA from total lung homogenates by RT-qPCR. (**E**) Lungs from mice infected with IAV PR8 LD were analyzed at day 1 (D1) and day 3 (D3) post-infection for the presence of DVGs and genomic fragments by PCR. Results for two different D3 mice are shown. Sequences for starred products can be seen in Fig. S8. (**F**) Expression of *Ifnb* mRNA in whole lung homogenates analyzed by RT-qPCR. Gene expression is shown as copy number relative to the housekeeping genes *Tuba1b* and *Rps11*. (*p<0.05, **p<0.01, ***p<0.001, ****p<0,0001; Unpaired, two tailed, t student test). Position of base pair size reference markers is indicated in each gel.

To investigate whether the content of DVGs in IAV stocks affects virulence similar to SeV, we obtained IAV strain PR8 stocks with a high content of DVGs (HD) or lacking DVGs (LD). The stock of IAV PR8 HD produced two predominant DVGs derived from the PA and PB1 genomic segments in infected cells, while no DVGs were detected in cells infected with IAV PR8 LD (Fig. 6A) (Strategy for IAV detection and sequences for the IAV DVGs present in infected cells can be found in Fig. S7). Mice infected with IAV PR8 HD showed reduced morbidity compared to mice infected with IAV PR8 LD (Fig. 6B) despite similar levels of virus replication (Fig. 6C). Similar to SeV Cantell HD, reduced morbidity was associated with enhanced *Ifnb* mRNA expression in the lung (Fig. 6D).

To determine whether IAV DVGs were generated *in situ* in the infected lung, we tracked their appearance in mice infected with IAV PR8 LD. Accumulation of DVGs was clearly observed at day 3 post-infection (Fig. 6E). Representative sequences of starred IAV DVGs products are shown in Fig. S8. Similar to SeV infection, accumulation of DVGs corresponded with enhanced expression of mRNA for *Ifnb* and *Il-6* (Fig. 6F) despite evidence of reduced genomic viruses at that time point (Figs. 6E and F). These data demonstrate that DVGs are generated *de novo* in the lung during infections with IAV, and suggest an important role of these types of genomes in promoting the host response to IAV *in vivo*.

## Discussion

We have shown that DVGs are naturally generated in the lung during infection with SeV and IAV and provide primary danger signals for the triggering of the host response to infection. The generation of DVGs during virus growth in tissue culture is a highly conserved phenomenon among viruses of different species and is tempting to speculate that DVGs provide an evolutionary advantage to the virus by contributing to the preservation of both the virus and the host. Interestingly, immunostimulatory DVGs result from drastic truncations in the genome of the virus that render it a dead end product unable to persist in the absence of helper virus. It will be relevant to determine how DVGs relate to viral “quasispecies” that result from mutations as a consequence of having a viral polymerase with a lower fidelity and processivity [37]. Viral quasispecies have been shown to be essential for viral fitness and virulence [37]. Whether DVGs a tradeoff of this viral polymerase characteristic that enables more rapid virus evolution but makes the virus more vulnerable to innate immune detection remains to be established.

Our data demonstrate that DVG recognition is not necessary for the response to NDV, while is required for the response to SeV. We speculate that the differential DVG requirement may be explained by the poor adaptation of the avian NDV to grow in mice while the murine SeV is fully adapted to grow in this species. A critical factor of this adaptation is the activity of the virally encoded V and C proteins that effectively block the induction of type I IFNs, as well as the type I IFN-mediated amplification of the type I IFN pathway [38], [39], [40], [41]. As NDV is adapted to grow in birds, its antagonistic proteins are not fully functional in mammalian cells [42] allowing unrestricted production and amplification of type I IFNs. In contrast, the SeV C and V proteins very effectively block the cellular response to SeV [40], [42], [43], [44] and no cellular response is observed unless DVGs are present. Interestingly, in the absence of type I IFN feedback (or of the IFN-inducible transcription factor IRF7) SeV DVGs induce a more potent cellular response compared to NDV, suggesting that DVGs have a unique ability to bypass both virus antagonism and the requirement for IRF7 for strong type I IFN production. We have reported that SeV Cantell HD, but not NDV, has the ability to induce the expression of the viral sensor MDA5 independently of type I IFN feedback [27], and that MDA5 is involved in the recognition of SeV DVGs ([23] and data in Fig. S4). Although it is unclear why this newly synthetized MDA5 is less susceptible to inhibition by the SeV V protein, preferential binding of DVGs to both RIG-I and MDA5 compared to standard SeV genomes, together with the availability of high levels of MDA5, may explain the strong activation of transcription factors and type I IFN expression in response to DVGs, regardless of type I IFN feedback.

Remarkably, DVGs arise *in vivo* independently of type I IFN feedback, demonstrating that DVGs do not appear in response to host pressure via type I IFNs. Notably, viruses containing DVGs have significantly reduced virulence. In a previous study, we reported that a SeV strain with a lower propensity to produce DVGs (SeV 52) persisted longer in the lung than a SeV strain able to produce high levels of DVGs (Cantell) [26]. Consistently, we observed higher levels of viral NP protein at day 7 post-infection in the lung of mice infected with SeV Cantell LD, compared to mice infected with SeV Cantell LD plus DPs (Fig. 4G). In additional studies, we have not observed significant differences in the rate of SeV-specific T cells in the lung of mice infected with SeV LD alone or in the presence of DPs (data not shown and [26]). Although interpretation of this observation is complicated by the reduced amount of SeV antigen (NP protein) present in infection with SeV LD plus DPs, we favor the hypothesis that DPs diminish virulence by competing for the viral polymerase, thus interfering with the replication of the standard virus, a well-defined characteristic of DVGs. Additionally, enhanced production of type I IFNs in response to DVGs likely contributes to dampened viral replication.

Highly immunostimulatory SeV DVGs are of the copy-back type. Intriguingly, during SeV infections *in vivo*, accumulation of DVGs of high molecular weight preceded the appearance of low molecular weight DVGs, suggesting that the smaller ones may be secondary to longer defective genomic products. Copy-back DVGs are not transcribed due to their promoter properties [10], thus their stimulatory activity likely derives solely from their genomic composition. IAV DVGs are truncated versions of one of the genomic segments that have natural complementarity among their 3′ and 5′ ends providing the theoretical capacity to form structures similar to copy-back DVGs. Notably, it is apparent that both SeV genomic and DVG RNAs have the potential to induce a host response when delivered naked into the cells. Based on our data, we predict that in the context of infection, DVG RNA is more available for detection due to their enhanced rate of replication compared to standard viral genomes (Fig. 2). Interestingly, we have shown that DVGs have the ability to bypass viral-encoded antagonists of the immune response even upon overexpression of viral antagonistic proteins [24]. It remains to be investigated what is the molecular mechanism behind this DVG property.

Notably, DVGs of different forms and compositions have been described in the sera of patients chronically infected with a number of different viruses [13], [14], [15], [16], [17] and DVGs of various viruses have been shown to promote persistent infections in tissue cultures [45], [46], [47], [48], [49], [50], [51], [52], [53] supporting a role for DVGs in the maintenance of chronic viruses. The role of naturally arising DVGs in promoting virus persistence *in vivo* remains to be investigated.

In summary, we have demonstrated that DVGs arise naturally during an acute respiratory virus infection and that they play a critical role in regulating the virus-host cycle *in vivo*. Importantly, the recognition of DVGs as stimuli for the onset of immunity has multiple practical implications, most directly: (i) DVGs represent novel determinants of virus pathogenesis that could be targeted for therapy, and (ii) DVGs are novel candidate biomarkers to predict the outcome of infections and the rate of virus spread in the population.

## Materials and Methods

### Ethics statement

This study was carried out in strict accordance with the recommendations in the Guide for the Care and Use of Laboratory Animals of the National Institutes of Health. The protocol (803176) was approved by the Institutional Animal Care and Use Committee, University of Pennsylvania Animal Welfare Assurance Number A3079-01.

### Cell lines and mice

TC-1 cells (mouse lung epithelial cells, ATCC, #DR-L2785), LLC-MK2 cells (monkey kidney cells, ATCC, #CCL7), MDCK (Madin-Darby canine kidney cells, kindly provided by Dr. T. Moran, Icahn School of Medicine at Mount Sinai), Baby hamster kidney-21 (BHK-21) cells expressing the T7 RNA polymerase (BSR-T7) (kindly provided by Dr. C. Basler, Icahn School of Medicine at Mount Sinai), and WT and *Ifnar1* ^−/−^ mouse embryo fibroblasts (kindly provided by Dr. B. tenOever, Icahn School of Medicine at Mount Sinai) were cultured in DMEM supplemented with 10% fetal bovine serum, 1 mM sodium pyruvate, 2 mL L-Glutamine, and 50 mg/ml gentamicin. C57BL/6 mice were obtained from Taconic Farms, Inc. IFNβ-YFP reporter mice (B6.129-*Ifnb1^tm1Lky^/J*) were obtained from The Jackson Laboratories. *Ifnar1* ^−/−^ mice were a kind donation of Dr. Thomas Moran (Icahn School of Medicine at Mount Sinai) and were bred in our animal facility.

### Viruses

SeV strains Cantell, 52, Enders, and Z, and influenza A/PR8/34 virus were grown in 10 days hen embryonated eggs (SPAFAS; Charles River Laboratories). SeV Cantell was passaged to retain its original high DI particle content (HD) or to deplete it of DI particles (LD) as we previously described [24]. In brief, SeV 52, Enders, Z, and Cantell HD were grown in embryonated hen eggs inoculated with 30,000 medium tissue culture infectious dose (TCID_50_) for 40 h. SeV Cantell HD TCID_50_ was calculated by end point dilution in LLCMK2 cells in the presence of trypsin, as described below [24]. SeV Cantell HD total particles were calculated by end point dilution of hemagglutination of chicken red blood cells. SeV Cantell HD stocks had consistently an infectious:total particle ratio of 5,000–15,000. SeV 52 had an infectious:total particle ratio of 24,472. SeV Enders had an infectious:total particle ratio of 38,746. SeV Z had an infectious:total particle ratio of 391,553. SeV Cantell LD was prepared by inoculating embryonated hen eggs with 3 TCID_50_ for 40 h. Under these conditions only 33% of the eggs grew virus. Allantoic fluid from those eggs was pooled and diluted 1/10^6^ for subsequent inoculation into embryonated hen eggs in a total volume of 100 µl. Allantoic fluid containing virus (80% of the inoculated eggs) was pooled and tittered as described below. SeV Cantell LD stocks had consistently an infectious:total particle ratio of 100,000–200,000. IAV strain PR8 (LD) was grown by inoculating hen embryonated eggs with 30,000 TCID_50_ obtained directly from infected lung homogenates. Allantoic fluid containing the virus was collected 40 h later. Egg's allantoic fluid was snap frozen in an ethanol/dry-ice bath and stored at −80°C. IAV PR8 containing high dose of DI particles (HD) was kindly provided by Dr. Laurence C. Eisenlohr, V.M.D., Ph.D (Thomas Jefferson University). IAV HD was grown by inoculating hen embryonated eggs with 10,000 pfu of egg-passed virus. Eggs were incubated at 35°C and allantoic fluid containing the virus was collected 48 h later. IAV PR8 HD and LD stocks were originated from the same parent stock but were extensively passaged in the different conditions described.

### Virus titrations

Permissive cells were infected with serial 1∶10 dilutions of lung homogenates or virus stocks in the presence of 2 mg/ml of trypsin to determine the medium tissue culture infectious dose (TCID_50_). LLCMK2 cells were used for SeV titration, while MDCK cells were used for IAV titration. After 72 h of incubation at 37°C, 50 µl of supernatant from each well was tested by hemagglutination of chicken red blood cells (RBCs) for the presence of virus particles at the end point dilution. To do this, 1∶4 dilutions of the cell supernatant were incubated in 0.5% chicken RBCs at 4°C for 30 min. Hemagglutination of RBCs indicated the presence of SeV or influenza virus particles.

### Generation of bone marrow-derived dendritic cells (BMDCs) and infection

BMDCs were generated as previously described [24]. Detailed procedure can be found in the Supplemental Information Material and Methods. BMDCs were infected after 4 days in culture with viruses at an multiplicity of 1.5 as we have previously described [24].

### Mice infections

For SeV infections, mice were anesthetized with tribromoethanol (Avertin®; Acros Organics) and inoculated in the nostrils with 30 µl of PBS containing 10^4^ or 10^5^ TCID_50_ of SeV. For IAV infections animals were infected intranasally with 100 TCID_50_/mouse in a 30 µl volume. Lungs were extracted at different times post-infection, homogenized in 0.1% w/v Gelatin-PBS and snap frozen in dry-ice/ethanol for preservation.

### RNA extraction and PCR for viral genome detection

Total RNA was extracted from cell lines or lungs with TRIzol (Invitrogen) according to the manufacturer's specifications and total RNA was reversed transcribed using the high capacity RNA to cDNA kit from Applied Biosystem. For sorted cells, 500 ng of RNA were reversed transcribed, for all other experiments 1–2 µg of RNA were reversed transcribed. cDNA was diluted to a concentration of 10 µg/µl and amplified with specific primers in the presence of SYBR green (Applied Biosystem). For the detection of DVGs, isolated total RNA was reverse transcribed using Superscript III without RNase H activity, to avoid self-priming by the DVGs complementary ends and recombinant RNase H (Invitrogen) was added later to the samples. For the detection of the standard virus genome, the negative strand of the full-length genome was reverse transcribed with Transcriptor First Strand cDNA synthesis kit (Roche). PCR detection for IAV was performed using as it has been previously described [54]. Primers and detailed PCR conditions can be found in the Supplemental Information Material and Methods.

### RT-qPCR

Detailed primers and PCR conditions can be seen in the Supplemental Information Material and Methods.

### Western blot

Whole cellular extracts were prepared by lysing 3×10^6^ of cells in a NP-40-based lysis buffer containing phosphatase inhibitors, proteinase inhibitors (Roche and Thermo Scientific), and 0.5 M EDTA. The concentration of protein was measured by Bradford assay (Themo Scientific). Samples (25 µg) were boiled for 5 min and resolved on 10% Bis-Tris pre-cast gels (Bio-rad). Resolved proteins were transferred to a polyvinylidene fluoride (PVDF) membrane (Millipore). The membrane was blocked with 5% non-fat milk and immunoblotted with the indicated antibodies. Anti-rabbit IRF3, anti-rabbit phospho-IRF3 (Ser396), anti-mouse IκBα, anti-mouse phospho-IκBα (Ser32/36), and anti-rabbit IgG (HRP-conjugated) were purchased from Cell Signaling. Anti-mouse GAPDH was purchased from Sigma. Anti-mouse IgG and anti-mouse IgG_1_ (HRP-conjugated) were purchased from Jackson Immunologicals. Lumi-Light western blotting substrate was used for HRP detection (Roche).

### SeV defective particles (DPs) purification

DP purification was performed as previously described [24]. In short, allantoic fluid from 100 infected hen eggs was pooled and concentrated by high-speed centrifugation. Pellets were suspended in 0.5 ml of PBS/2 mM EDTA and incubated overnight at 4°C in a 5–45% sucrose (Fisher) gradient that was prepared using a gradient maker (BioComp). Gradients were centrifuged at 4°C for 1.5 h at 28,000 rpm and fractions containing low-density viral particles were collected, pelleted, suspended and re-purified using the same procedure. Collected low-density fractions were concentrated by centrifugation at 4°C for 2 h at 21,000 rpm. Pellets were suspended in PBS, snap frozen, and stored at −80°C. The content of DI particles was determined by calculating the ratio of infectious over non-infectious particles as described above.

### Rescue of recombinant virus

A 591 nt long product containing the sequence of the T7 promoter followed by the 546-nucleotide long copy back DVG from SeV Cantell, and flanked by the restriction enzymes SpeI and SapI at the 3′ an 5′ ends was synthetically synthetized (DNA 2.0) and clone into the pSL1180 vector (Amersham Pharmacia Biotech) containing the sequences for the hepatitis delta virus ribozyme and the T7 polymerase terminator. In order to optimize the transcription of the DVG, 3 G residues were introduced downstream of the T7 promoter by site-directed mutagenesis (Stratagene, CA) using the oligonucleotides 5′CCACTAGTTAATACGACTCACTATA**GGG**ACCAGACAAGAGTTTAAGAG-3′ and 5′CTCTTAAACTCTTGTCTGGT**CCC**TATAGTGAGTCGTATTAACTAGTGG-3′. BSR-T7 cells were infected with a moi of approximately 66 of partially inactivated SeV strain 52. Virus inactivation was performed by exposing diluted virus to UV light (254 nm model MRL-58, UVP Upland, CA) for 53 sec at a distance of 9 inches from the light source. Virus inactivation diminished the virus replication rate, while allowing the expression of viral proteins necessary for the replication of DVGs. Cells were incubated at 37°C for 1 h before transfection of 3 µg of vector encoding DVG. Transfection was performed with XtremeGENE transfection reagent (Roche) according to manufacturer instructions. Cells were cultured in Dulbecco's modified Eagle medium (DMEM) supplemented with 1% bovine serum albumin, 2% NaCO_3_, 0.5 µg/ml trypsin (Worthington) and 0.1% penicillin-streptomycin (Invitrogen) and incubated in 7% CO_2_ at 37°C. Cells and supernatant containing SeV 52 and rDPs were harvested after 48 h and 200 µl of the suspension were inoculated in the allantoic cavity of 10-day embryonated hen eggs (B & E Eggs, Silver Springs, PA). After 40 h allantoic fluid was harvest and 200 µl of undiluted fluid were inoculate in 10-day embryonated eggs for virus growth and egg inoculation was repeated for three consecutive passages. Allantoic fluid from the third passage was quick-frozen in dried ice/ethanol and used for infections. Presence of recombinant DVG was confirmed by PCR. No other DVGs were detected.

### RNA treatments

DVG RNA was *in vitro* transcribed (Ambion) from the T7-DVG 546 plasmid. Standard genomic RNA was extracted from SeV Cantell LD stocks. To remove 5′-triphosphates, 1 µg of RNA was incubated with 10 U of Calf Intestinal Phosphatase (New England BioLabs) for 60 min at 37°C. To cleave single stranded RNA, 1 µg of RNA was incubated with 1 ng of RNase A (Ambion) for 15 min at room temperature. To cleave double stranded RNA, RNA was incubated with 0.1 U of RNase V1 (Ambion) for 15 min at room temperature. After treatments, RNA was purified using TRIzol or precipitation/inactivation buffer according to the manufacturer's specifications.

### RNA transfection

LLC-MK2 cells were transfected with 250 ng or indicated doses of DVG and genomic RNA using lipofectamin 2000 (invitrogen). At 4 hours post transfection, the cells were harvested and total RNA was isolated using TRIzol according to the manufacturer's specifications.

### Flow cytometry and sorting

Infected IFNβ-YFP cells were collected at 6 h post-infection. Reporter mice were sacrificed 3 days post-infection. Lungs were collected and dissociated with collagenase (Roche), followed by suspension on 0.5 M EDTA and RBC lysis buffer. Single cell suspensions were then incubated with CD16/CD32 FcBlock for 20 min at 4C, followed by incubation with biotinylated mouse anti-CD45.2. Washed cells were incubated with anti-biotin microbeads (Miltenyi) for 20 min and passed through a magnetic column for negative selection. CD45^−^ cells were sorted based on YFP expression using a FACS Vantage SE sorter.

### Statistical analysis

Statistical analyses were performed as indicated in each figure. GraphPad Prism version 5.00 for Windows, GraphPad Software, San Diego California USA, www.graphpad.com, was used for analysis.

**Genes NCBI ID numbers.** *tuba1b*: 22143; *rps11:27207; ifnb: 15977; ifnl2:* 330496; *illb: 16176; il12b*: 16160; *tnf: 2926; Il-6*, 16193.

## Supporting Information

Figure S1 **SeV copy-back DVG PCR strategy and validation.** (**A**) Diagram of the genomic composition of the full length SeV genome (gSeV) and of a representative copy-back DVG of unknown length. Arrows indicate the location of primers used for RT and amplification (PCR). Full-length size of the genome is indicated. Expected amplicon size of 760 nt of the gSeV to be detected through our PCR assay is indicated. This strategy allows detection of most copy-back DVGs replicating in an infected cell. (**B**) Schematics of SeV strain Cantell's predominant 546 nt-long DVG. Expected amplicon size of 278 nt of this particular DVG to be detected through our PCR assay is indicated. (**C**) Validation of the DVG PCR assay. DVG and gSeV amplicons from plasmids encoding the full length SeV strain Cantell genome (lane 1) or the SeV strain Cantell dominant DVG (lane 2) after amplification using the primers depicted in (A).
(TIF)

Figure S2 **Potent expression of** ***Ifnb*** **mRNA in response to DVGs is independent of IRFs 1, 5, 7, and 8.** BMDCs were prepared from WT, *Irf1^−/−^*, *Irf3^−/−^*, *Irf5^−/−^*, *Irf7^−/−^*, and *Irf8^−/−^* mice. The cells were infected with SeV Cantell HD or SeV Cantell LD (moi = 1.5 TCID_50_/cell) and harvested 2 h post-infection to determine the induction of *Ifnb* expression in total RNA by RT-qPCR. Gene expression is shown as copy number relative to the housekeeping genes *Tuba1b* and *Rps11*. Error bars indicate the standard deviation of triplicate measurements in a representative experiment. Asterisks indicate values that were statistically significant (***, p<0.001, unpaired t-test). ns means the result is statistically insignificant.
(TIF)

Figure S3 **DVGs efficiently trigger the host response in different cell types.** (**A**) TC-1 cells, NIH3T3 cells, RAW264.7 cells, and BMDCs were infected with SeV Cantell HD or SeV Cantell LD (moi = 1.5 TCID_50_/cell). Phosphorylation of IRF3 was determined from whole cell extracts 2 and 6 h post-infection. (**B**) Expression of SeV *Np* mRNA in TC-1 cells and BMDCs detected by RT-qPCR. Gene expression is shown as copy number relative to the housekeeping genes *Tuba1b* and *Rps11*. Error bars indicate the standard deviation of triplicate measurements in a representative experiment. Asterisks indicate values that were statistically significant (*, p<0.05, **, p<0.01 unpaired t-test). ns means the result is statistically insignificant.
(TIF)

Figure S4 **DVGs stimulate MAVS-dependent activation of transcription factors and cytokine expression.** (**A**) BMDCs generated from WT and *Mavs^−/−^* mice were infected with SeV Cantell HD, SeV Cantell LD (moi = 1.5 TCID_50_/cell), or HSV-1 (moi = 5 TCID_50_/cell). Whole cellular extracts from mock treated or infected cells were prepared at the indicated time points. The presence of unphosphorylated and phosphorylated IRF3 and IκBα, and MAVS were examined by western blot. Content of GAPDH was used as loading control. (**B**) Total RNA was isolated from infected WT or *Mavs^−/−^* BMDCs at 6 h post-infection and the expression of type I IFN, pro-inflammatory cytokines, and viral gene mRNAs was analyzed by RT-qPCR. (**C**) RAW 264.7 cells were infected with SeV HD or SeV LD (moi = 3 TCID_50_/cell). After 6 h, total cellular protein was extracted and immunoprecipitated with anti-RIG-I or anti-MDA5 antibodies. RNA eluted from the immunoprecipitation was tested for gSeV and DVGs by PCR. (**D**) SeV *Np* and *Ifnb* mRNA was quantitated by RT-qPCR. (**E**) Primary lung fibroblasts prepared form WT or MDA5 KO mice were infected with a moi of 1 TCID_50_/cell SeV Cantell HD and analyzed for gene expression at 6 h after infection. (**F**) WT or RIG-I KO MEFs mice were infected with a moi of 1.5 TCID_50_/cell of SeV Cantell HD and analyzed for gene expression at 6 h after infection. Gene expression is shown as copy number relative to the housekeeping genes *Tuba1b* and *Rps11*. Error bars indicate the standard deviation of triplicate measurements in a representative experiment. Asterisks indicate values that were statistically significant (***, p<0.001, *, p<0.05 unpaired t-test). ns means the result is statistically insignificant.
(TIF)

Figure S5 **Representative sequences of SeV DVGs arising in infected cells.** (**A**) Sequence of a low molecular weight DVG that arises during infection with moi = 1.5 TCID_50_/cell SeV Cantell LD. DVG (546) shows the sequence of a reference SeV Cantell DVG of 546 bp. (**B**) Sequence of the amplicon of a high molecular weight DVG found during the infection with SeV Cantell LD. (**C**) Sequence of the amplicon of the lowest molecular weight DVGs that arises during an infection with SeV Z. All these sequences refer to Fig. 2 of the manuscript.
(TIF)

Figure S6 **Copy-back DVGs are generated in the lung during infection with SeV 52.** C57BL6 mice were infected with 10^4^ TCID_50_/mouse (10 ID_50_) of SeV 52 and total RNA from lungs was analyzed for copy-back DVGs and standard viral genomes (gSeV) by PCR. Position of base pair size reference markers is indicated in each gel.
(TIF)

Figure S7 **IAV DVG PCR design and sequences of DVGs present in IAV strain PR8 stocks.** (**A**) Diagram of the single step PCR strategy used for the detection of IAV genomic segments. Predicted fragment sizes are indicated (adapted from Ref. [54]) (**B**) Alignment of the IAV PA segment with a DVGs fragment of 482 bp present in the IAV HD stock. (**C**) Alignment of the IAV PB1 segment with a DVGs fragment of 430 bp present in the IAV HD stock. Primer sequences are boxed. These sequences refer to Fig. 5 of the manuscript.
(TIF)

Figure S8 **Representative sequences of IAV defective viral genomes generated** ***in vivo*** **.** (**A**) Alignment of the IAV PA segment with a DVG fragment of 390 bp cloned from lungs infected with IAV PR8 for three days. (**B**) Alignment of the IAV PB1 segment with a DVG fragment of 398 bp cloned from lungs infected with IAV PR8 for three days. Primer sequences are boxed. These sequences refer to Fig. 5 of the manuscript.
(TIF)

Text S1 **Supporting** **materials and methods** **.** The text includes in detail descriptions of the procedures, as well as specific material and methods and references for figures included as supporting information.
(DOCX)
