# Supplementary figures and images for: Defective Viral Genomes Arising In Vivo Provide Critical Danger Signals for the Triggering of Lung Antiviral Immunity
Source: PLoS Pathog. 2013 Oct 31;9(10):e1003703. doi: 10.1371/journal.ppat.1003703 (PMC3814336; doi:10.1371/journal.ppat.1003703)

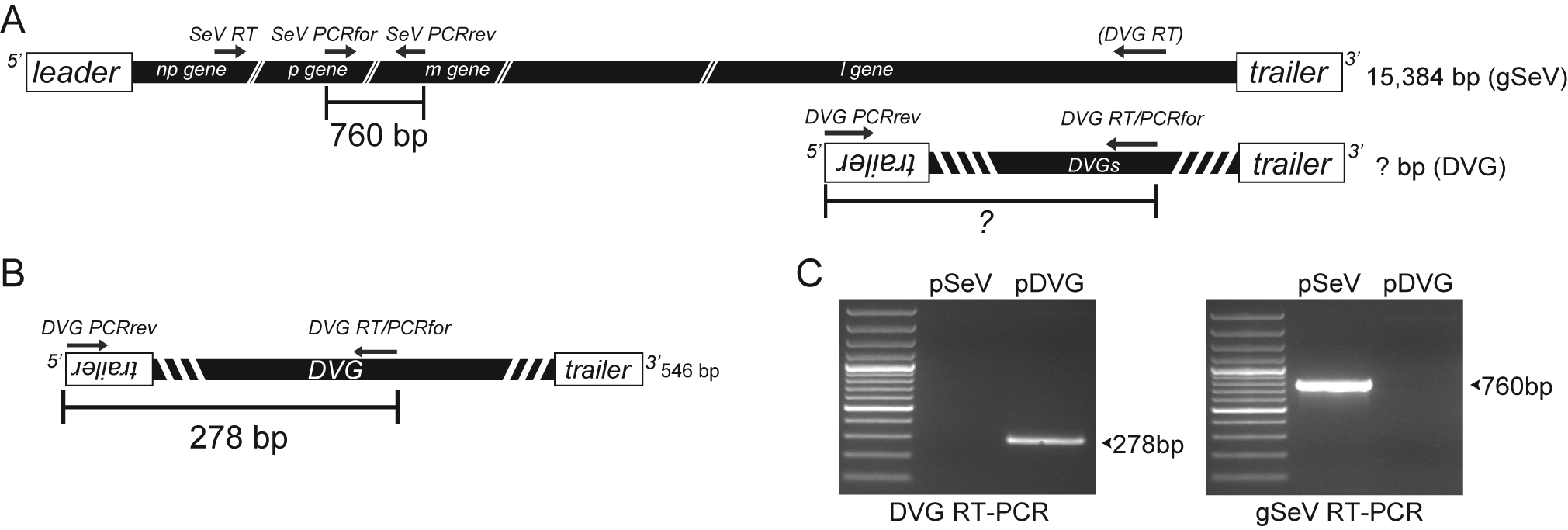

Supplement: Figure S1 — SeV copy-back DVG PCR strategy and validation. (A) Diagram of the genomic composition of the full length SeV genome (gSeV) and of a representative copy-back DVG of unknown length. Arrows indicate the location of primers used for RT and amplification (PCR). Full-length size of the genome is indicated. Expected amplicon size of 760 nt of the gSeV to be detected through our PCR assay is indicated. This strategy allows detection of most copy-back DVGs replicating in an infected cell. (B) Schematics of SeV strain Cantell's predominant 546 nt-long DVG. Expected amplicon size of 278 nt of this particular DVG to be detected through our PCR assay is indicated. (C) Validation of the DVG PCR assay. DVG and gSeV amplicons from plasmids encoding the full length SeV strain Cantell genome (lane 1) or the SeV strain Cantell dominant DVG (lane 2) after amplification using the primers depicted in (A). (TIF) [file ppat.1003703.s001.tif]

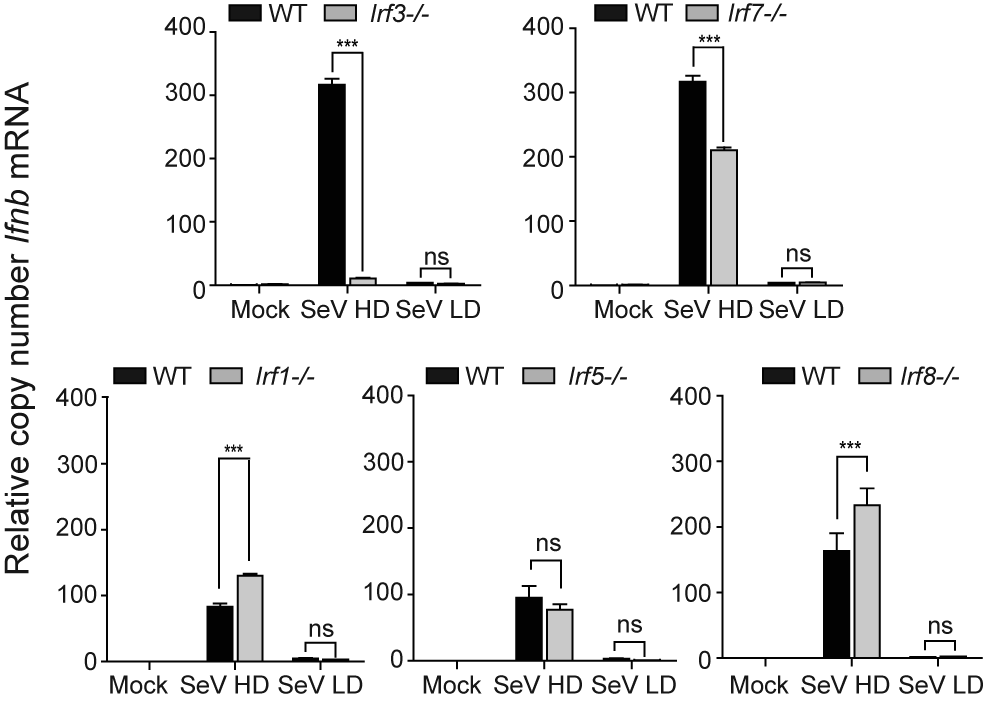

Supplement: Figure S2 — Potent expression of Ifnb mRNA in response to DVGs is independent of IRFs 1, 5, 7, and 8. BMDCs were prepared from WT, Irf1−/−, Irf3−/−, Irf5−/−, Irf7−/−, and Irf8−/− mice. The cells were infected with SeV Cantell HD or SeV Cantell LD (moi = 1.5 TCID50/cell) and harvested 2 h post-infection to determine the induction of Ifnb expression in total RNA by RT-qPCR. Gene expression is shown as copy number relative to the housekeeping genes Tuba1b and Rps11. Error bars indicate the standard deviation of triplicate measurements in a representative experiment. Asterisks indicate values that were statistically significant (***, p<0.001, unpaired t-test). ns means the result is statistically insignificant. (TIF) [file ppat.1003703.s002.tif]

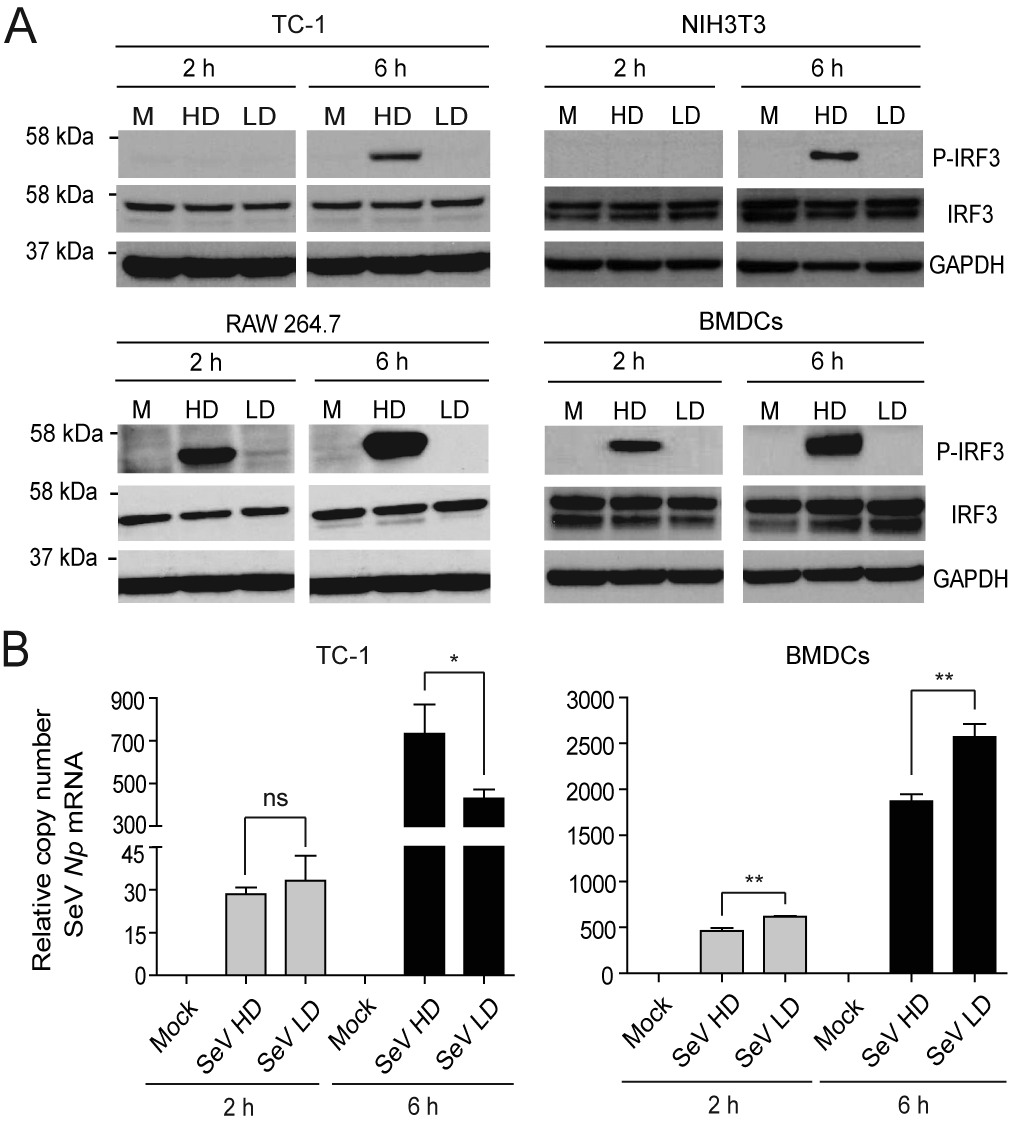

Supplement: Figure S3 — DVGs efficiently trigger the host response in different cell types. (A) TC-1 cells, NIH3T3 cells, RAW264.7 cells, and BMDCs were infected with SeV Cantell HD or SeV Cantell LD (moi = 1.5 TCID50/cell). Phosphorylation of IRF3 was determined from whole cell extracts 2 and 6 h post-infection. (B) Expression of SeV Np mRNA in TC-1 cells and BMDCs detected by RT-qPCR. Gene expression is shown as copy number relative to the housekeeping genes Tuba1b and Rps11. Error bars indicate the standard deviation of triplicate measurements in a representative experiment. Asterisks indicate values that were statistically significant (*, p<0.05, **, p<0.01 unpaired t-test). ns means the result is statistically insignificant. (TIF) [file ppat.1003703.s003.tif]

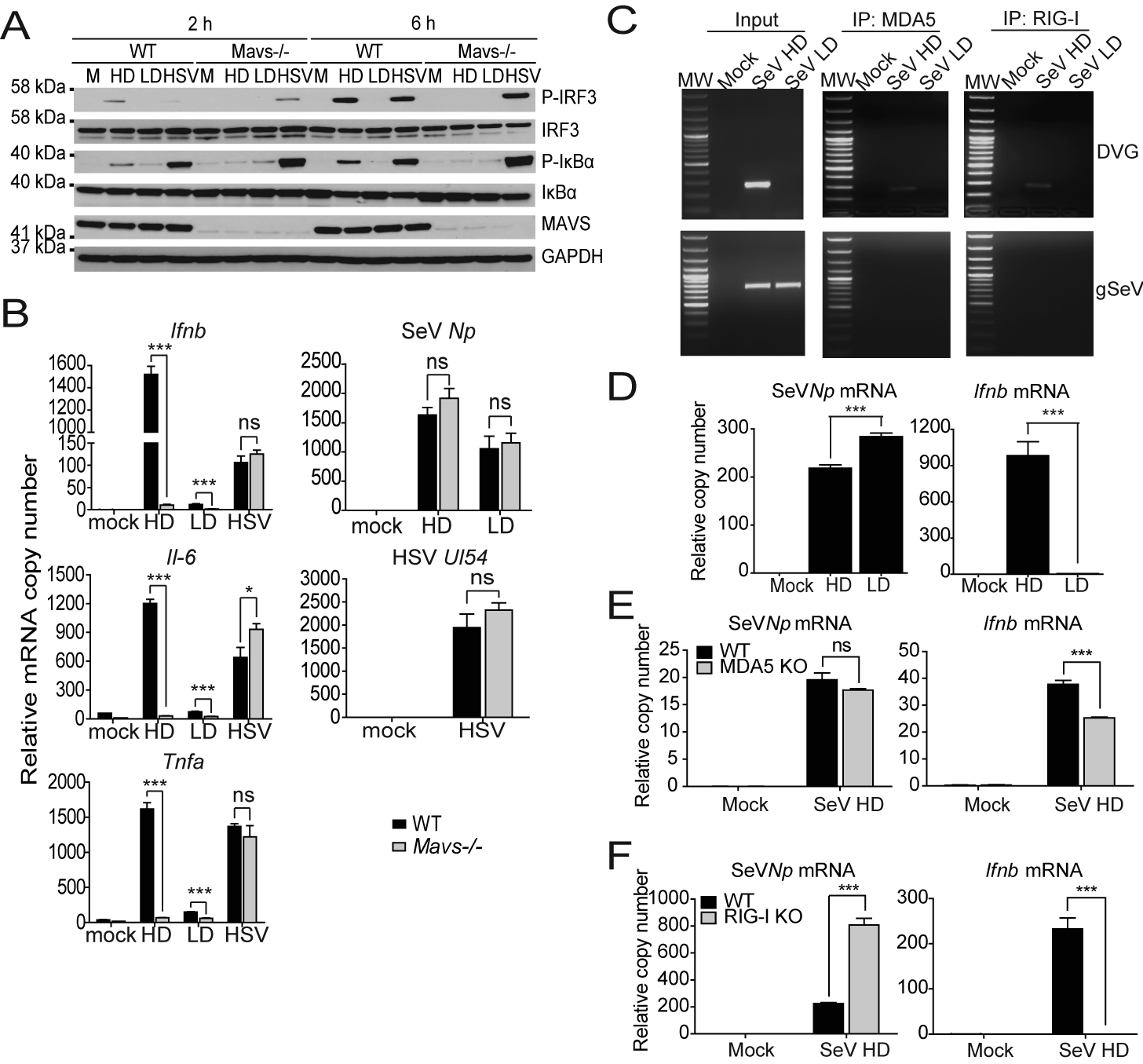

Supplement: Figure S4 — DVGs stimulate MAVS-dependent activation of transcription factors and cytokine expression. (A) BMDCs generated from WT and Mavs−/− mice were infected with SeV Cantell HD, SeV Cantell LD (moi = 1.5 TCID50/cell), or HSV-1 (moi = 5 TCID50/cell). Whole cellular extracts from mock treated or infected cells were prepared at the indicated time points. The presence of unphosphorylated and phosphorylated IRF3 and IκBα, and MAVS were examined by western blot. Content of GAPDH was used as loading control. (B) Total RNA was isolated from infected WT or Mavs−/− BMDCs at 6 h post-infection and the expression of type I IFN, pro-inflammatory cytokines, and viral gene mRNAs was analyzed by RT-qPCR. (C) RAW 264.7 cells were infected with SeV HD or SeV LD (moi = 3 TCID50/cell). After 6 h, total cellular protein was extracted and immunoprecipitated with anti-RIG-I or anti-MDA5 antibodies. RNA eluted from the immunoprecipitation was tested for gSeV and DVGs by PCR. (D) SeV Np and Ifnb mRNA was quantitated by RT-qPCR. (E) Primary lung fibroblasts prepared form WT or MDA5 KO mice were infected with a moi of 1 TCID50/cell SeV Cantell HD and analyzed for gene expression at 6 h after infection. (F) WT or RIG-I KO MEFs mice were infected with a moi of 1.5 TCID50/cell of SeV Cantell HD and analyzed for gene expression at 6 h after infection. Gene expression is shown as copy number relative to the housekeeping genes Tuba1b and Rps11. Error bars indicate the standard deviation of triplicate measurements in a representative experiment. Asterisks indicate values that were statistically significant (***, p<0.001, *, p<0.05 unpaired t-test). ns means the result is statistically insignificant. (TIF) [file ppat.1003703.s004.tif]

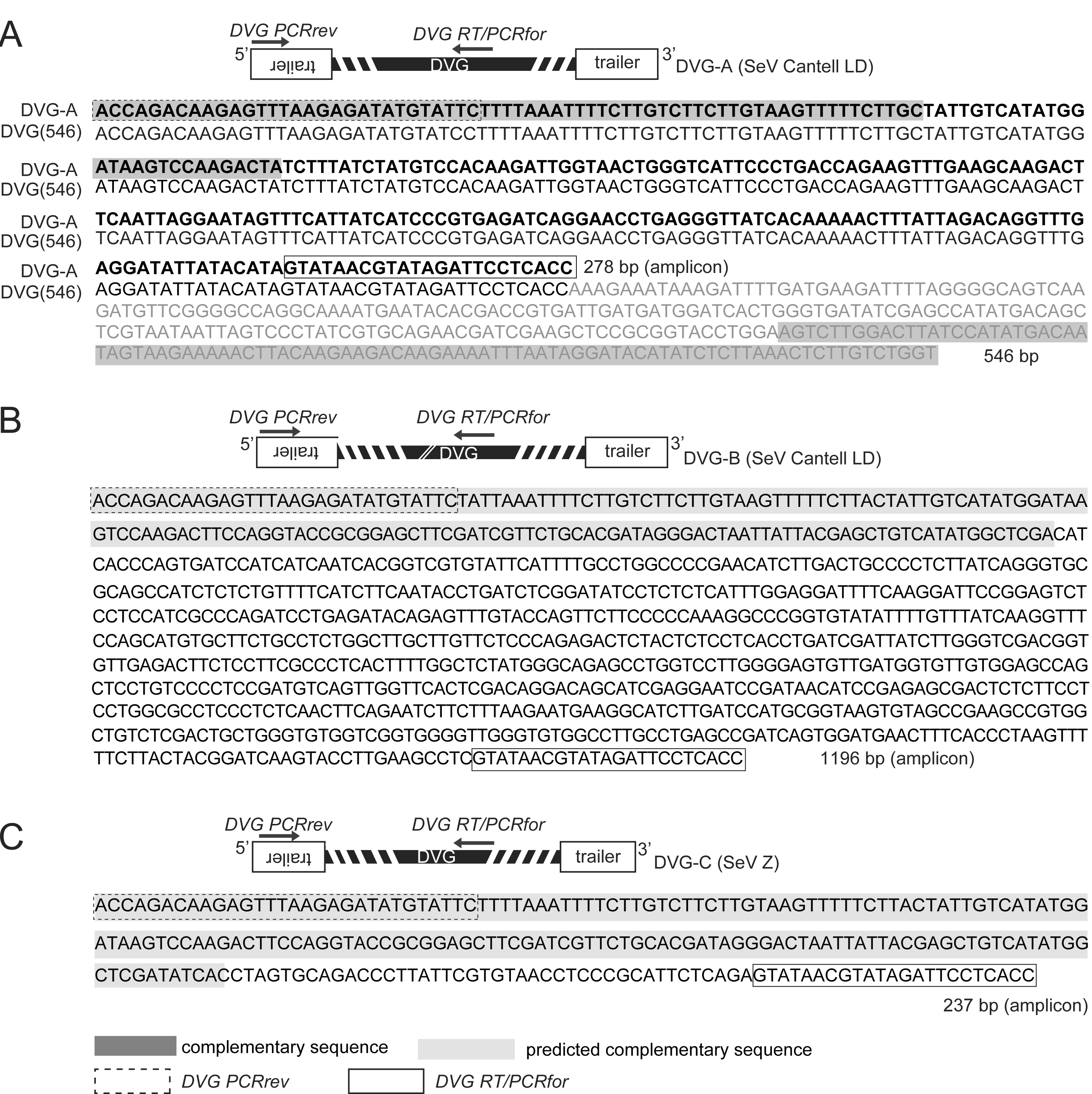

Supplement: Figure S5 — Representative sequences of SeV DVGs arising in infected cells. (A) Sequence of a low molecular weight DVG that arises during infection with moi = 1.5 TCID50/cell SeV Cantell LD. DVG (546) shows the sequence of a reference SeV Cantell DVG of 546 bp. (B) Sequence of the amplicon of a high molecular weight DVG found during the infection with SeV Cantell LD. (C) Sequence of the amplicon of the lowest molecular weight DVGs that arises during an infection with SeV Z. All these sequences refer to Fig. 2 of the manuscript. (TIF) [file ppat.1003703.s005.tif]

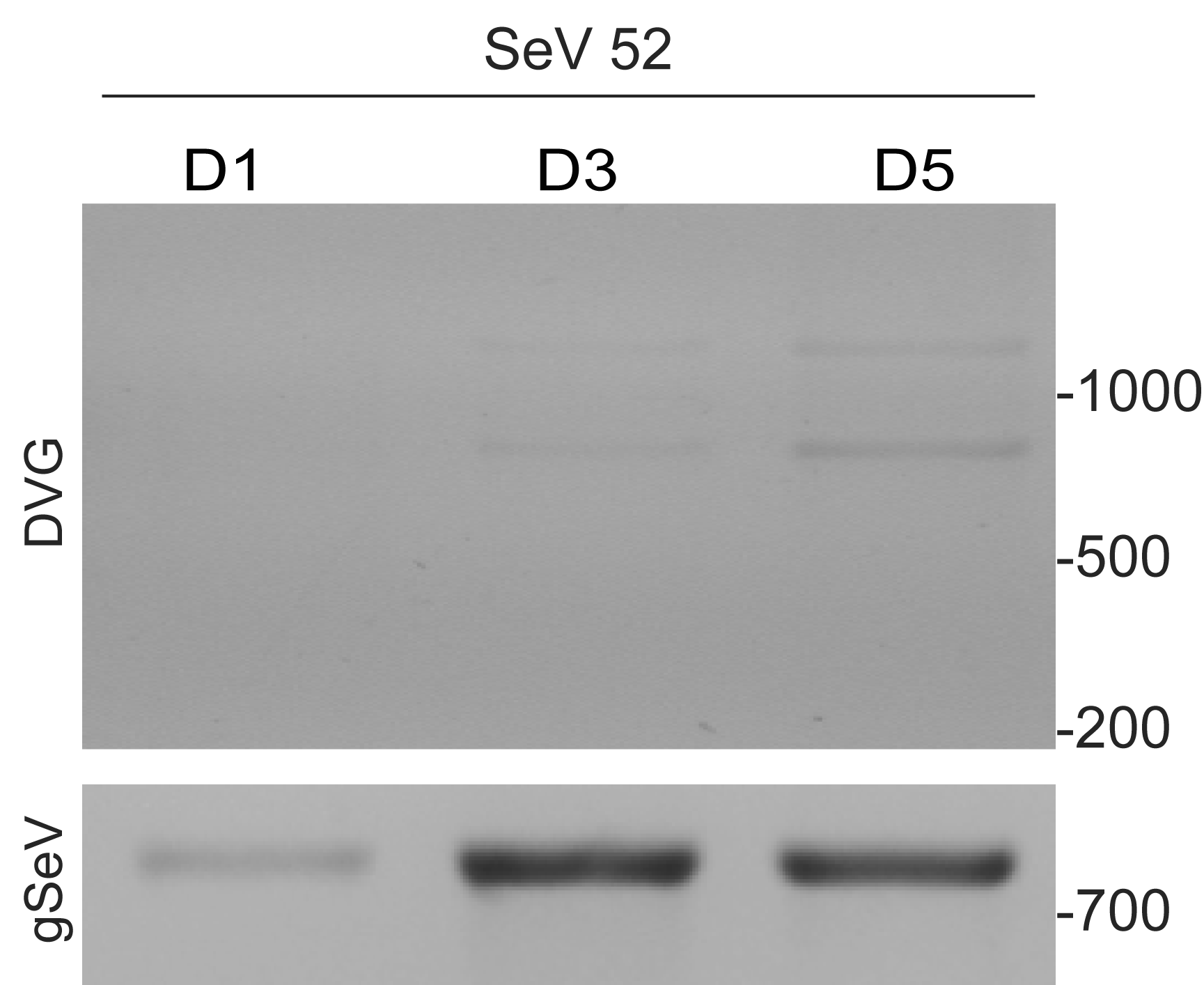

Supplement: Figure S6 — Copy-back DVGs are generated in the lung during infection with SeV 52. C57BL6 mice were infected with 104 TCID50/mouse (10 ID50) of SeV 52 and total RNA from lungs was analyzed for copy-back DVGs and standard viral genomes (gSeV) by PCR. Position of base pair size reference markers is indicated in each gel. (TIF) [file ppat.1003703.s006.tif]

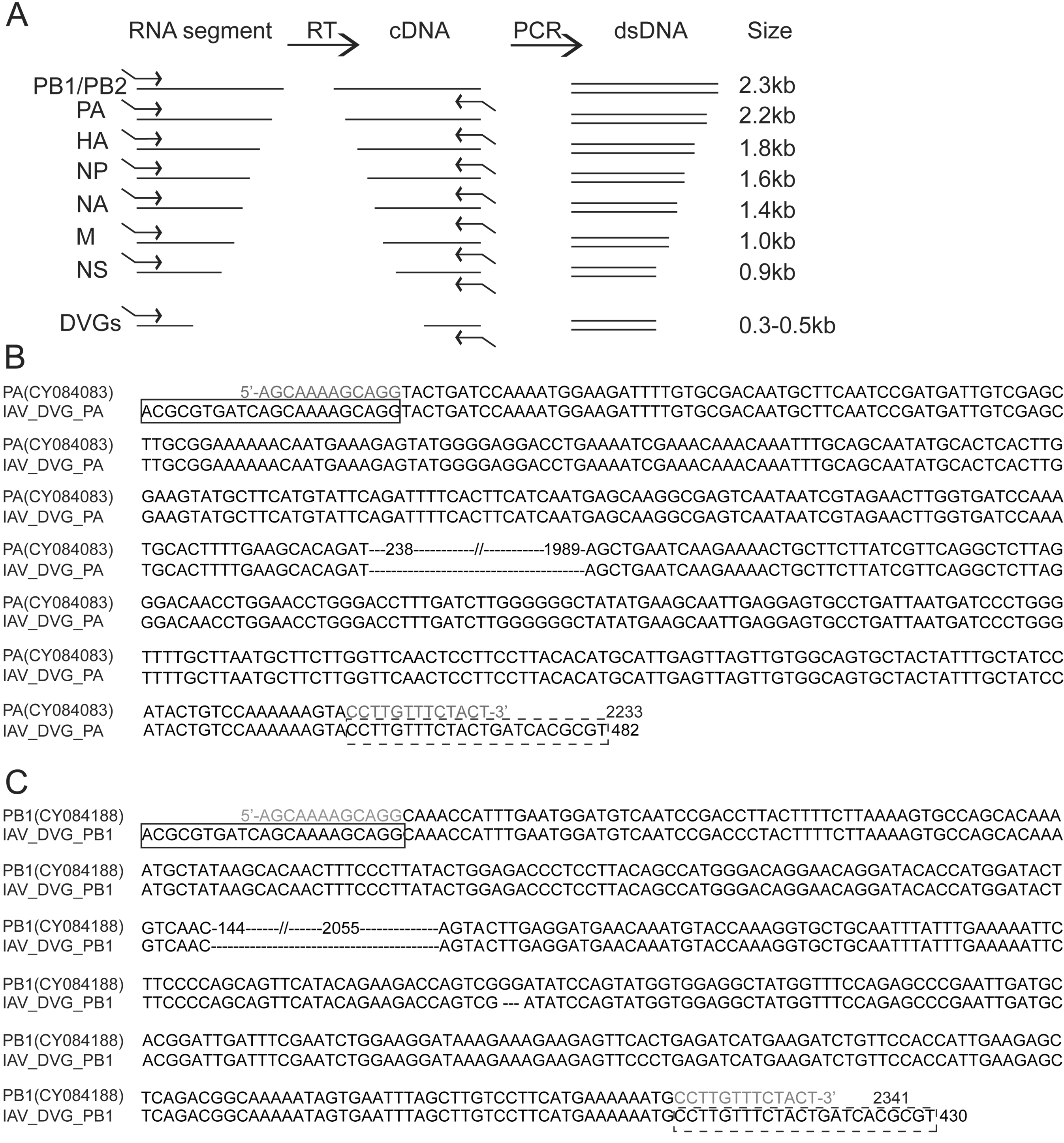

Supplement: Figure S7 — IAV DVG PCR design and sequences of DVGs present in IAV strain PR8 stocks. (A) Diagram of the single step PCR strategy used for the detection of IAV genomic segments. Predicted fragment sizes are indicated (adapted from Ref. [54]) (B) Alignment of the IAV PA segment with a DVGs fragment of 482 bp present in the IAV HD stock. (C) Alignment of the IAV PB1 segment with a DVGs fragment of 430 bp present in the IAV HD stock. Primer sequences are boxed. These sequences refer to Fig. 5 of the manuscript. (TIF) [file ppat.1003703.s007.tif]

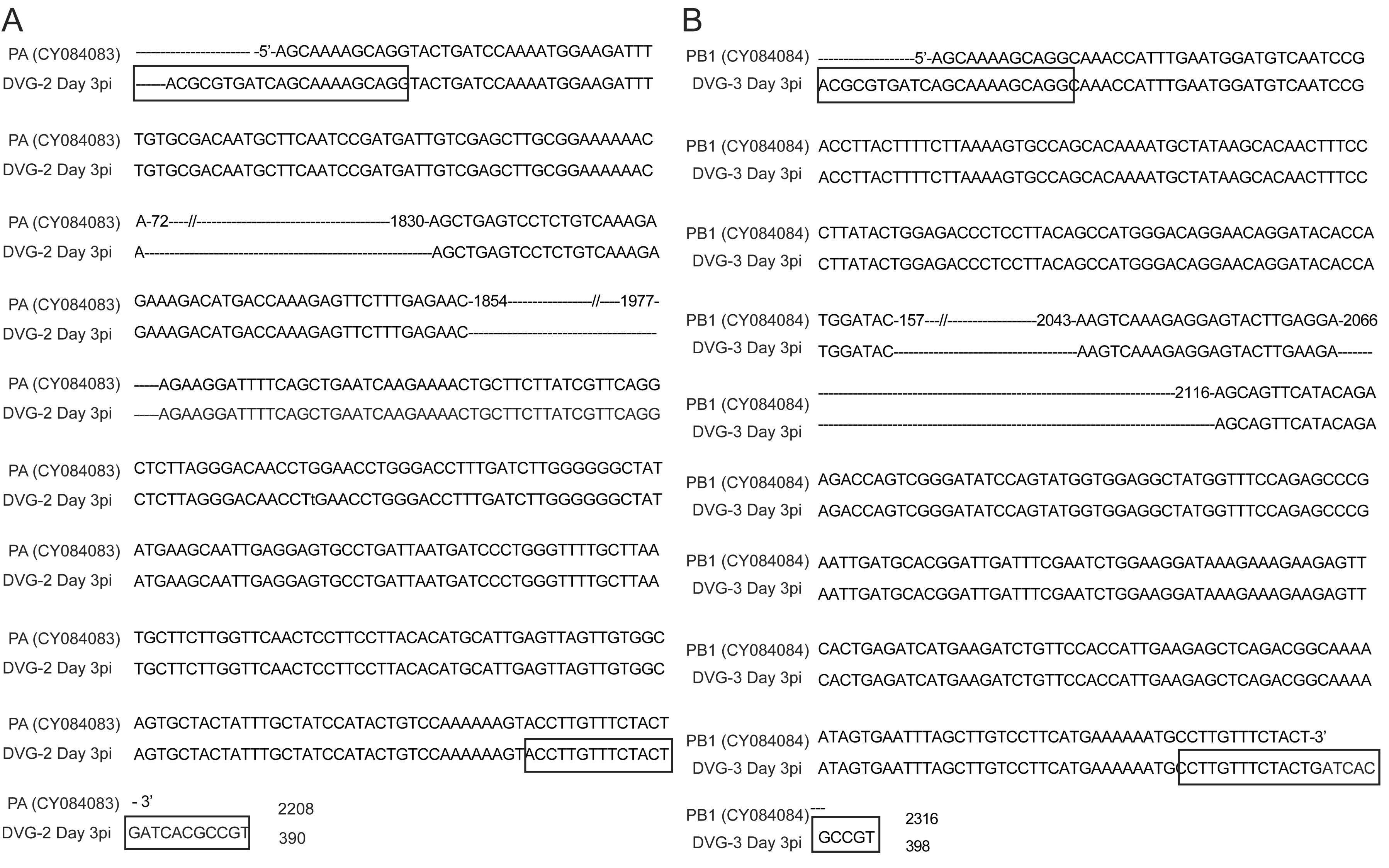

Supplement: Figure S8 — Representative sequences of IAV defective viral genomes generated in vivo . (A) Alignment of the IAV PA segment with a DVG fragment of 390 bp cloned from lungs infected with IAV PR8 for three days. (B) Alignment of the IAV PB1 segment with a DVG fragment of 398 bp cloned from lungs infected with IAV PR8 for three days. Primer sequences are boxed. These sequences refer to Fig. 5 of the manuscript. (TIF) [file ppat.1003703.s008.tif]
