# Supplementary material for: Defective Viral Genomes Arising In Vivo Provide Critical Danger Signals for the Triggering of Lung Antiviral Immunity
Source: PLoS Pathog. 2013 Oct 31;9(10):e1003703. doi: 10.1371/journal.ppat.1003703 (PMC3814336; doi:10.1371/journal.ppat.1003703)
Supplement: Text S1 — Supporting materials and methods . The text includes in detail descriptions of the procedures, as well as specific material and methods and references for figures included as supporting information. (DOCX) [file ppat.1003703.s009.docx]

**Supporting Information**

**SI Materials and Methods**

**Additional Viruses**

Herpes Simplex virus 1 (HSV) strain KOS 1.1 was propagated and tittered by limiting dilution on Vero cells as previously described [[1](#_ENREF_1)].

**Generation of bone marrow-derived dendritic cells (BMDCs) and infection**

BMDCs were generated as previously described [[2](#_ENREF_2)] from C57BL/6, *Irf1^-/-^,* *Irf3^-/-^*, *Irf5^-/-^, Irf7^-/-^*, *Irf8^-/-^,* and *Mavs^-/-^* bone marrow. Mice and/or bones were kindly provided by Drs. Katherine Fitzgerald (University of Massachusetts, MA), Michael S. Diamond (Washington University, MO), Paula M. Pitha-Rowe (Johns Hopkins University, MD), Huobao Xiong (Icahn School of Medicine at Mount Sinai, NY), or commercially obtained from The Jackson Laboratory (*Irf1^-/-^*). Briefly, bone marrow from femur and tibias was flushed, red blood cells were lysed, and lymphocytes and MHC class II^+^ cells were depleted using rat anti-CD8 (Clone 53-6.7), anti-CD4 (clone GK1.5), anti-B220/CD45R (clone RA3-6B2), and anti-IA/IE (clone 2G9) antibodies (BD Biosciences) followed by negative selection using magnetic beads coated with anti-rat IgG (Polysciences Inc.). BMDCs precursors were plated at 7x10^5^ cells/ml in RPMI medium containing 1% syngeneic NMS, 50 mg/ml gentamycin, 2 mM L-Glutamine, 1 mM NaPy and 25 U/ml murine GM-CSF (PeproTech Inc.). Cells were infected after 4 days in culture.

**Murine lung fibroblast preparation**

Lungs were digested in DMEM containing 0.28 wU/ml of collagenase (Liberase TM grade, Roche) for 1 hour at 37℃ followed by incubation with 0.5 mM EDTA for 5 min. Cells were filtrated through a 70 um strainer and plated at a concentration of 1x10^7^ cells/well in a 6 well plate. The cells were used for experiments at day 7 post-culture.

**Primers and PCR conditions**

1. **PCR for SeV DVG and gSeV detection:**

For detection of DVGs, total RNA was reverse transcribed with the primer 5’GGTGAGGAATCTATACGTTATAC3’ using SuperScript III reverse transcriptase without RNase H activity to avoid self-priming. Recombinant RNase H (Invitrogen) was later added to the reverse transcribed samples and incubated for 20 min at 37°C. cDNA was amplified using the primers: *for*-5’GGTGAGGAATCTATACGTTATAC3’ and *rev*-5’ACCAGACAAGAGTTTAAGAGATATGTATT3’. For detection of standard viral genome, RNA was reverse transcribed with Transcriptor First Strand cDNA synthesis kit (Roche) using the primer 5’TGTTCTTACTAGGACAAG3’. Amplification of the intergenomic segment between the *p* and *m* genes was performed using the primers: *for*-5’AATCTAGGTATCTCACTCCATG3’ and *rev*-5’AAGAGATTCTCGAGTATCAGAA3’. The temperature cycle parameters used for the PCR in a BioRad C1000 Thermal Cycler were: 95°C for 10 min and 33-35 cycles of 95°C for 45 sec, 55°C for 30 sec and 72°C for 90 sec followed by a hold at 72°C for 5 min.

**b. PCR for IAV DVG and gIAV detection:**

PCR detection for IAV was performed using the primers MBTuni-12 5′-ACGCGTGATCAGCAAAAGCAGG and MBTuni-13 5′-ACGCGTGATCAGTAGAAACAAGG as it has been previously described [[3](#_ENREF_3)].

**c. RT-qPCR for mRNA quantitation:**

Equivalent amounts of RNA from each sample (0.5-2.0 μg) were reverse transcribed using the High Capacity RNA-to cDNA Kit (Applied Biosystems). cDNA was diluted to a concentration of 10 μg/μl in distilled water. qPCR reactions were performed in triplicate using specific primers and the Power SYBR® Green PCR Master Mixture (Applied Biosystems) in a Viia7 Applied Biosystem Lightcycler. Normalization was conducted based on levels of *Tuba1b* and *Rps11*. The following primer sequences were used: *tuba1b: for*-5’TGCCTTTGTGCACTGGTATG3’, *rev*-5’CTGGAGCAGTTTGACGACAC3’; *rps11: for*-5’CGTGACGAAGATGAAGATGC3’, *rev*-5’GCACATTGAATCGCACAGTC3’; *ifn-β for*-5’AGATGTCCTCAACTGCTCTC3’, *rev*-5’AGATTCACTACCAGTCCCAG3’; *ifn- *: *for*-5’AGGTCTGGGAGAACATGACTG3’, *rev*-5’CTGTGGCCTGAAGCTGTGTA3’; *il-1β for*-5’TTGACGGACCCCAAAAGAT3’, *rev*-5’GATGTGCTGCTGCGAGATT3’; *il-6* *for*-5’ ACAGAAGGAGTGGCTAAGGA3’, *rev*-5’ CGCACTAGGTTTGCCGAGTA3’; *il-12p40*: *for*-5’TTGAAAGGCTGGGTATCGGT3’, *rev*-5’GAATTTCTGTGTGGCACTGG3’; *tnf*-*α * *for*-5’TCACTGGAGCCTCGAATGTC3’, *rev*-5’GTGAGGAAGGCTGTGCATTG3’; *sev np* *for*-5’TGCCCTGGAAGATGAGTTAG3’, *rev*-5’GCCTGTTGGTTTGTGGTAAG3’; *hsv ul54*: *for*-5’CCCTTTCTCCAGTGCTACCT3’, *rev*-5’CTCTCCGACCCCGACACCAA3’; *ndv hn*: *for*-5’CAATGCTGAGACAATAGGTC3’, *rev*-5’GACAATGCTTGATGGTGAAC3’; *flu np*: *for*-5’CAGCCTAATCAGACCAAATG3’, *rev*- 5’TACCTGCTTCTCAGTTCAAG3’.

**d. RT-qPCR for gSeV and SeV DVG quantitation:**

cDNA was synthesized from 2 μg of total RNA using SuperScript III First-Strand RT synthesis system from Invitrogen according to the manufacturer’s protocol. Recombinant RNase H (Invitrogen) was later added to the reverse transcribed samples and incubated for 20 min at 37°C. cDNA was then diluted 1 to 20. Genomic and DVG RT-qPCR reactions were performed in a total volume of 10 μl in the presence of 4 μl of diluted cDNA, 5 μl of Power SYBR® Green PCR Master Mixture (Applied Biosystems), and 1 μl of primer mixture as follows: DVG, *for* 5’-GGTGAGGAATCTATACGTTATAC-3’, *rev* 5’-ACCAGACAAGAGTTTAAGAGATATGTATT-3’; gSeV, *for* 5’-TGCCCTGGAAGATGAGTTAG-3’, *rev* 5’-GCCTGTTGGTTTGTGGTAAG-3’. All Ct values were normalized to the housekeeping gene *Rps11*, which was quantified as described above. The RT-qPCR assay was validated using a plasmid expressing the full-length gSeV or the full length DVG-546 genome.

**Immunoprecipitation**

RAW 264.7 cells were infected with SeV HD or SeV LD (moi=3 TCID_50_/cell). After 6 h, total cellular protein was extracted using an NP-40-based lysis buffer containing RNase inhibitors (Invitrogen), phosphatase inhibitors (Roche), proteinase inhibitors (Roche), and 0.5 M EDTA (Thermo Scientific). The concentration of protein was measured using Bradford assay (Themo Scientific). The samples (1 mg) were pre-cleared by treatment with 30 μl of protein-A coated magnetic beads (Milipore) for 30 min at 4°C. The cellular extract was then incubated with a primary antibody (1μg) specific for RIG-I or MDA5 (Cell signaling, clones D14G6 and D74E4 respectively) for 2 h at 4°C followed incubation with protein A magnetic beads (Milipore) for 2 h. The immunocomplexes were washed 4 times using high salt buffer containing 500 mM NaCl, 50 mM Tris-HCl (pH 8.0), 1% NP-40 in 50ml distilled water, followed by one wash with low salt buffer containing 20 mM Tris-HCl (pH 7.5) in 50ml of distilled water. RNA was eluted from the immunoprecipitation with elution buffer containing 100 mM Tris-Cl (pH 8.0), 10 Mm EDTA, 1 % SDS, RNase inhibitors, and proteinase inhibitors.

**Cloning and sequencing**

Fragments obtained from DVG-PCR were cloned in pGemT-Easy vector (Promega), according to the manufacturer’s protocol and used to transform XL10 Gold ultracompetent cells (Stratagene). Cells were plated in LB Agar EZMix (Sigma,) and then growth in LB Broth EZMix (Sigma). Plasmid DNA was extracted using QIAprep Miniprep (Quiagen). Plasmid DNA was sequenced in 454 GS FLX Sequencer (University of Pennsylvania DNA Core facility). Sequences were analyzed with Lasergene software.

**SI References**

1. Cotter CR, Kim WK, Nguyen ML, Yount JS, Lopez CB, et al. (2011) The virion host shutoff protein of herpes simplex virus 1 blocks the replication-independent activation of NF-kappaB in dendritic cells in the absence of type I interferon signaling. Journal of virology 85: 12662-12672.

2. Yount JS, Kraus TA, Horvath CM, Moran TM, Lopez CB (2006) A novel role for viral-defective interfering particles in enhancing dendritic cell maturation. J Immunol 177: 4503-4513.

3. Zhou B, Donnelly ME, Scholes DT, St George K, Hatta M, et al. (2009) Single-reaction genomic amplification accelerates sequencing and vaccine production for classical and Swine origin human influenza a viruses. Journal of virology 83: 10309-10313.
